# The neuroanatomical substrates of autism and ADHD and their link to putative genomic underpinnings

**DOI:** 10.1186/s13229-023-00568-z

**Published:** 2023-10-04

**Authors:** Lisa M. Berg, Caroline Gurr, Johanna Leyhausen, Hanna Seelemeyer, Anke Bletsch, Tim Schaefer, Charlotte M. Pretzsch, Bethany Oakley, Eva Loth, Dorothea L. Floris, Jan K. Buitelaar, Christian F. Beckmann, Tobias Banaschewski, Tony Charman, Emily J. H. Jones, Julian Tillmann, Chris H. Chatham, Thomas Bourgeron, The EU-AIMS LEAP Group, Jumana Ahmad, Sara Ambrosino, Bonnie Auyeung, Simon Baron-Cohen, Sarah Baumeister, Sven Bölte, Carsten Bours, Michael Brammer, Daniel Brandeis, Claudia Brogna, Yvette de Bruijn, Bhismadev Chakrabarti, Ineke Cornelissen, Daisy Crawley, Flavio Dell’Acqua, Guillaume Dumas, Sarah Durston, Jessica Faulkner, Vincent Frouin, Pilar Garcés, David Goyard, Lindsay Ham, Hannah Hayward, Joerg Hipp, Rosemary Holt, Mark H. Johnson, Prantik Kundu, Meng-Chuan Lai, Xavier Liogier D’Ardhuy, Michael V. Lombardo, David J. Lythgoe, René Mandl, Andre Marquand, Luke Mason, Maarten Mennes, Andreas Meyer-Lindenberg, Carolin Moessnang, Nico Bast, Laurence O’Dwyer, Marianne Oldehinkel, Bob Oranje, Gahan Pandina, Antonio M. Persico, Barbara Ruggeri, Amber Ruigrok, Jessica Sabet, Roberto Sacco, Antonia San José Cáceres, Emily Simonoff, Will Spooren, Roberto Toro, Heike Tost, Jack Waldman, Steve C. R. Williams, Caroline Wooldridge, Marcel P. Zwiers, Declan G. Murphy, Christine Ecker

**Affiliations:** 1 Department of Child and Adolescent Psychiatry, University Hospital, Goethe University, Deutschordenstrasse 50, 60528 Frankfurt am Main, Germany; 2 Brain Imaging Center, Goethe University, 60528 Frankfurt am Main, Germany; 3 Department of Biosciences, Goethe University Frankfurt, 60438 Frankfurt am Main, Germany; 4 Fries Lab, Ernst Strüngmann Institute (ESI) for Neuroscience in Cooperation with Max Planck Society, 60528 Frankfurt, Germany; 5 Department of Forensic and Neurodevelopmental Sciences, Institute of Psychiatry, Psychology and Neuroscience, King’s College, London, SE5 8AF UK; 6 Department of Cognitive Neuroscience, Donders Institute for Brain, Cognition and Behaviour, Radboud University Nijmegen Medical Center, Nijmegen, The Netherlands; 7 Methods of Plasticity Research, Department of Psychology, University of Zurich, Zurich, Switzerland; 8 Child and Adolescent Psychiatry, Medical Faculty Mannheim, Central Institute of Mental Health, University of Heidelberg, Mannheim, Germany; 9 Department of Psychology, Institute of Psychiatry, Psychology and Neuroscience, King’s College London, London, SE5 8AF UK; 10 Centre for Brain and Cognitive Development, Birkbeck, University of London, Malet Street, London, WC1E 7JL UK; 11 F. Hoffmann–La Roche, Innovation Center Basel, Basel, Switzerland; 12 Human Genetics and Cognitive Functions Unit, Institut Pasteur, Paris, France; 13 Department of Psychology, Social Work and Counselling, Faculty of Education and Health, Greenwich University, London, UK; 14 University Medical Center Utrecht, Utrecht University, Utrecht, Netherlands; 15 School of Philosophy, Psychology and Language Sciences, University of Edinburgh, 7 George Square, Edinburgh, EH8 9JZ UK; 16 Autism Research Centre, Department of Psychiatry, University of Cambridge, Cambridge, UK; 17 Center of Neurodevelopmental Disorders (KIND), Centre for Psychiatry Research, Department of Women’s and Children’s Health, Karolinska Institutet and Stockholm Health Care Services, Region Stockholm, Stockholm, Sweden; 18 Child and Adolescent Psychiatry, Stockholm Health Care Services, Region Stockholm, Stockholm, Sweden; 19 Curtin Autism Research Group, Curtin School of Allied Health, Curtin University, Perth, Australia; 20 Department of Child and Adolescent Psychiatry and Psychotherapy, Psychiatric Hospital, University of Zurich, Zurich, Switzerland; 21 Neuroscience Center Zurich, University and ETH Zurich, Zurich, Switzerland; 22 Pediatric Neurology Unit, Università Cattolica del Sacro Cuore, 00168 Rome, Italy; 23 Neurospin Centre CEA, Gif sur Yvette, France; 24 Roche Pharma Research and Early Development, Neuroscience, Ophthalmology and Rare Diseases, Roche Innovation Center Basel, Basel, Switzerland; 25 Regulatory Affairs, Pharmaceutical Development, F. Hoffmann-La Roche Pharmaceuticals, Basel, Switzerland; 26 Department of Radiology, Icahn School of Medicine at Mount Sinai, New York, NY USA; 27 Child and Youth Mental Health Collaborative, Centre for Addiction and Mental Health and The Hospital for Sick Children, Department of Psychiatry, University of Toronto, Toronto, Canada; 28 Laboratory for Autism and Neurodevelopmental Disorders, Center for Neuroscience and Cognitive Systems @UniTn, Istituto Italiano di Tecnologia, Rovereto, Italy; 29 Department of Neuroimaging, Institute of Psychiatry, Psychology and Neuroscience, King’s College London, London, UK; 30 Janssen Research and Development, Titusville, NJ USA; 31 Child and Adolescent Neuropsychiatry, Department of Biomedical, Metabolic and Neural Sciences, University of Modena and Reggio Emilia, Modena, Italy; 32 Social, Genetic and Developmental Psychiatry Centre, Institute of Psychiatry, Psychology and Neuroscience, King’s College London, London, UK; 33 Department of Child and Adolescent Psychiatry, Institute of Psychology, Psychiatry and Neuroscience, King’s College London, London, UK

**Keywords:** ASD, ADHD, Neurodevelopmental disorders, Comorbidity, Imaging-genetics, Structural MRI

## Abstract

**Background:**

Autism spectrum disorders (ASD) are neurodevelopmental conditions accompanied by differences in brain development. Neuroanatomical differences in autism are variable across individuals and likely underpin distinct clinical phenotypes. To parse heterogeneity, it is essential to establish how the neurobiology of ASD is modulated by differences associated with co-occurring conditions, such as attention-deficit/hyperactivity disorder (ADHD). This study aimed to (1) investigate between-group differences in autistic individuals with and without co-occurring ADHD, and to (2) link these variances to putative genomic underpinnings.

**Methods:**

We examined differences in cortical thickness (CT) and surface area (SA) and their genomic associations in a sample of 533 individuals from the Longitudinal European Autism Project. Using a general linear model including main effects of autism and ADHD, and an ASD-by-ADHD interaction, we examined to which degree ADHD modulates the autism-related neuroanatomy. Further, leveraging the spatial gene expression data of the Allen Human Brain Atlas, we identified genes whose spatial expression patterns resemble our neuroimaging findings.

**Results:**

In addition to significant main effects for ASD and ADHD in fronto-temporal, limbic, and occipital regions, we observed a significant ASD-by-ADHD interaction in the left precentral gyrus and the right frontal gyrus for measures of CT and SA, respectively. Moreover, individuals with ASD + ADHD differed in CT to those without. Both main effects and the interaction were enriched for ASD—but not for ADHD-related genes.

**Limitations:**

Although we employed a multicenter design to overcome single-site recruitment limitations, our sample size of *N* = 25 individuals in the ADHD only group is relatively small compared to the other subgroups, which limits the generalizability of the results. Also, we assigned subjects into ADHD positive groupings according to the DSM-5 rating scale. While this is sufficient for obtaining a research diagnosis of ADHD, our approach did not take into account for how long the symptoms have been present, which is typically considered when assessing ADHD in the clinical setting.

**Conclusion:**

Thus, our findings suggest that the neuroanatomy of ASD is significantly modulated by ADHD, and that autistic individuals with co-occurring ADHD may have specific neuroanatomical underpinnings potentially mediated by atypical gene expression.

**Supplementary Information:**

The online version contains supplementary material available at 10.1186/s13229-023-00568-z.

## Introduction

Autism spectrum disorders (ASD) are highly heterogeneous neurodevelopmental conditions characterized by differences in social interaction and communication, alongside restricted, repetitive behaviors and interests [1]. These features are associated with a different development of the brain [2] and regional differences in neuroanatomy [3, 4]. Yet, neuroanatomical differences in autism are highly variable across individuals and likely underpin different clinical phenotypes.

Autism is a condition with a high prevalence of co-occurring conditions. More specifically, 72% autistic adolescents have at least one co-occurring psychiatric disorder, and often meet diagnostic criteria for two or more co-occurring conditions [5]. Among these, attention-deficit/hyperactivity disorder (ADHD) is the most prevalent, with estimates ranging between 16 and 80% of autistic individuals also having a clinical diagnosis of ADHD [5–8]. The large range in prevalence estimates has mainly been attributed to variability in diagnostic methods employed [5, 6, 8]. Given the large degree of co-occurrence between autism and ADHD, it is likely that inter-individual differences in the neuroanatomy of autism are confounded by the severity and nature of ADHD features and so contribute to the highly complex clinical and neurobiological phenotype that is characteristic for autism. Overall, autism is characterized by neuroanatomical differences in several large-scale neural systems that include fronto-temporal and fronto-parietal regions, the amygdala-hippocampal complex, the cerebellum, basal ganglia, and anterior and posterior cingulate regions [2–4]. Many of these brain regions overlap with core components of the so-called social brain network, which comprises a set of brain regions that mediate functions related to social cognition and/or emotional processing [9]. Similarly, ADHD has been linked to neuroanatomical differences in frontal regions (e.g., dorsolateral and ventromedial prefrontal cortex), the parietal cortex, cingulate cortices, basal ganglia, the limbic lobe (e.g., amygdala and hippocampus) and the cerebellum [10–12]. There is thus a large degree of overlap between the neural networks underpinning autism and ADHD. Even though the neural systems subserving autism and ADHD seem to overlap to a large degree, neuroanatomical differences between both conditions have also been reported. For example, the neuroanatomical differences in the temporal lobe are less commonly reported in ADHD than in autism, while differences in cingulate brain regions seem to be more common in ADHD than autism. Moreover, it has been reported that individuals with ADHD (without co-occurring autism) have significantly reduced gray matter (GM) volume in fronto-temporal areas relative to autistic individuals, as well as significantly increased volume in parietal areas [13]. Another study reported that, compared controls, autistic individuals show increased cortical thickness (CT), while individuals with ADHD show for example decreased CT across the brain [14]. Taken together, these studies suggest that ASD and ADHD may have separable neuroanatomical underpinnings.

So far, only few studies have explored the neuroanatomy of autism with co-occurring ADHD. A study by Mizuno et al. [15] reported that autistic individuals with co-occurring ADHD have significantly decreased volume in the left postcentral gyrus compared to typically developing controls [15]. However, as this study only examined individuals with a diagnosis of both autism and ADHD relative to neurotypical controls, it was not possible to disentangle the relative impact of autism and ADHD on the level of brain anatomy. Another study observed significant differences in gray matter volume and surface area (SA) in the post- and precentral gyrus when comparing individuals with autism to individuals with autism and ADHD [16]. Further, there are reports of significant ASD-by-ADHD interactions in the parietal, temporal and limbic lobe for measures of CT, and in the right cingulate cortex for measures of cortical volume [17]. However, in this study, the interaction terms did not survive multiple comparison correction [17]. Neuroanatomical variability may therefore exist not only between neurodevelopmental conditions, but also between distinct measures of neuroanatomy.

Traditional investigations of brain structure have mainly focused on cortical volume, measured on the whole-brain, regional, and/or vertex-level. However, cortical volume is, by definition, the product of two different cortical features, namely CT and SA. Previous research by our group has shown that measures of brain volume, therefore, do not characterize a specific (i.e., unique) aspect of the neural architecture, but can be explained by separable variations in CT and SA (e.g., [18]). These two morphometric features also have distinct genetic determinants and developmental trajectories [19–22]. Thus, to characterize neuroanatomical variability associated with ASD and/or ADHD, it is important to investigate CT and SA separately. Using data provided by the EU-AIMS Longitudinal European Autism Project (LEAP; [23]), a large-scale European research collaboration on autism, we therefore examined the neuroanatomical underpinnings of autism with and without co-occurring ADHD symptomatology based on measures of CT and SA. Given the conceptual complexity of both ASD and ADHD [24–26], we utilized a categorical rather than a dimensional design that allowed us to establish the extent to which ADHD symptoms modulate the neuroanatomy of autism.

Moreover, leveraging the spatial gene expression data provided by the Allen Human Brain Atlas (AHBA; [27]) we aimed at linking these neuroanatomical differences to putative molecular underpinnings. Overall, neuropsychiatric conditions are highly heritable with an estimated heritability of 83% for autism [28], and 75% for ADHD [29]. However, the genetic architecture of autism is complex, involving hundreds (or more) genetic variants that mediate wider autism traits [30]. Many of these genes map onto biological pathways underpinning neural development [30–32]. The genetic architecture of ADHD is equally complex, and implicates mechanisms underpinning early embryonic development and cognitive abilities [33]. Previous studies also suggest that there is a significant genetic overlap between autism and ADHD. Overall, the genetic correlation between both conditions is 0.37 [34], which is higher than the genetic correlation between autism and other psychiatric disorders (e.g., *r* = 0.22 for autism and schizophrenia (SCZ), *r* = 0.14 for autism and bipolar disorder; [34]). Genome-wide association studies (GWAS) report several single nucleotide polymorphisms (SNPs) that are significantly associated with autism or ADHD, and a significant overlap between the SNPs associated with autism and those associated with ADHD has been reported [35]. These SNPs map onto genes that are predominantly expressed in the brain, and play a crucial role in neuronal migration, neuronal development, neuromodulation of neurotransmission, and in general brain development [35]. In the present study, we therefore also examined whether brain regions where the neuroanatomy of autism is significantly modulated by ADHD are enriched for genes that have previously been linked to autism and/or ADHD on the genetic and transcriptomic level.

## Methods and materials

### Participants

This study utilized data provided by the EU-AIMS LEAP project [23], which is a European multicenter study on stratification biomarkers for autism (www.aims-2-trials.eu). A comprehensive description of the sample has been published elsewhere [23]. In brief, the total sample for which usable structural MRI data were available included *N* = 638 individuals (*N* = 359 with ASD and *N* = 279 non-autistic controls). For the present study, a subset of *N* = 533 individuals aged between 6 and 30 years was selected that included all individuals with available data from the ADHD DSM-5 rating scale [1] (Table 1). Based on autism diagnostic criteria according to DSM-IV [36], DSM-IV-TR [37], DSM-5 [1], or ICD-10 [38] and whether participants met criteria for ADHD according to the ADHD DSM-5 rating scale [1], we subdivided the autistic individuals into two subgroups: ASD only (*N* = 170; 112 males, 58 females) and ASD with co-occurring ADHD [further referred to as ASD + ADHD group (*N* = 142; 107 males, 35 females)]. From the neurodiverse control group without an ASD diagnosis, we collected those individuals who met criteria for ADHD as a third subgroup [further referred to as ADHD only group (*N* = 25; 14 males, 11 females)] and those who did not meet ADHD criteria as our fourth subgroup [further referred to as typically developing (TD) controls (*N* = 196; 124 males, 72 females)]. The ADHD DSM-5 rating scale was based on either parent- or self-report scores depending on participants’ age. For this study we used the categorical output of the ADHD rating scale, which measures the presence of symptoms, evaluated on a 0–3 scale (0 = not at all to 3 = very often). To be evaluated with a clinical concern, a participant must at least score six responses with a 2 or 3 (“Often”, “Very Often”) (for more details see [23]). Therefore, the rating scale output is not a clinical diagnosis per se but the symptom count is a proxy for one. Notably, subsets were not matched for age, IQ, and sex to maximize the available sample size. We therefore controlled for these measures in all subsequent analyses (see below). Given the wide range in full scale IQ (FSIQ) across individuals, we also repeated our analyses in a smaller subset of individuals, which excluded participants with a mild intellectual disability (ID, i.e., FSIQ < 70). The results of these analyses are presented in the Additional file 1: Effects of Intellectual Disability. For further details on demographics and exclusion criteria see Additional file 1: Sample Description. An independent ethics committee at each site approved the study, and written informed consent was obtained for all participants.

**Table 1:** Characteristics of participants with ASD, ADHD, ASD + ADHD and control subjects

Variable	ASD only (*n* = 170)		ADHD only (*n* = 25)		ASD + ADHD (*n* = 142)		TD (*n* = 196)		Analysis		
	*N*	%	*N*	%	*N*	%	*N*	%	*Χ*^2^	*df*	*p*
Sex									7.18	3	0.07
Male	112	65.9	14	56	107	75.4	124	63.3			
Female	58	34.1	11	44	35	24.6	72	36.7			

	Mean	SD	Mean	SD	Mean	SD	Mean	SD	*F*	*df*	*p*
Age (years)	18.45	5.6	17.80	4.9	16.38	5.30	17.19	5.9	3.812	3	0.01**
FSIQ	101.98	18.8	79.8	21.1	94.93	20.8	108.44	14.1	28.29	3	< 2e−16***
Mean CT (mm)	2.67	0.12	2.67	0.13	2.69	0.14	2.68	0.11	1.07	3	0.36
Total SA (m^2^)	0.21	0.26	0.21	0.29	0.23	0.23	0.23	0.23	5.734	3	< 0.001***

Ages range from 7 to 30 years in the ASD only group, from 10 to 30 years in the ADHD only group, from 7 to 29 years in the ASD + ADHD group and from 6 to 30 years in the typical developing group. Full-scale IQ ranged from 58 to 148 in the ASD only group, from 50 to 119 in the ADHD only group, from 40 to 142 in the ASD × ADHD group and from 69 to 142 in the typical developing group

### MRI data acquisition

All participants underwent MR imaging in 3-T scanners, at six different sites (University of Cambridge and King’s College London, U.K.; Central Institute for Mental Health, Mannheim, Germany; Radboud University Medical Centre and University Medical Centre Utrecht, the Netherlands; Rome University, Italy). High-resolution structural T1-weighted volumetric images were acquired with full head coverage, at 1.2 mm thickness with 1.2 × 1.2-mm in-plane resolution (see Additional file 1: Table S1 for details).

### Cortical surface reconstructions using FreeSurfer

Usable structural MRI data were initially available for 708 individuals in the LEAP sample. FreeSurfer v6.0.0 software (http://surfer.nmr.mgh.harvard.edu/) was used to derive models of the cortical surface for each T_1_-weighted image. These well-validated and fully automated procedures have been previously described elsewhere [39–43]. Each reconstructed surface underwent strict quality assessments (see Additional file 1: MRI Data Quality Assessments), resulting in a final sample of *N* = 638 individuals [44]. In brief, three independent raters judged the quality of each scan with three possible decisions: include, exclude, edit [44]. In our study, we examined measures of CT and SA. Measures of CT were computed as the closest distance from the outer (i.e., pial) to the inner (i.e., white matter) boundary at each vertex on the tessellated surface [43]. Measures of surface area were quantified as the area of the cortex at a given point on the cortical surface (i.e., the sum of faces in the polygon mesh representation of the cortex at a particular vertex) as previously described by Winkler et al. [45]. For each participant we also computed mean CT across the entire brain (CT_0_), as well as total SA (SA_total_). To improve detection of population changes, each parameter was smoothed using a 15-mm surface based smoothing kernel.

### Surface-based statistical analyses of cortical thickness and surface area

Statistical analyses were performed using the SurfStat toolbox (https://www.math.mcgill.ca/keith/surfstat/) for MATLAB version R2021a (https://www.mathworks.com), and R for Statistical Computing (www.r-project.org). Vertex-wise differences in neuroanatomy (Y) were quantified using a general linear model (GLM) with (1) ASD, ADHD, sex, and site (see Additional file 1: Site effects for further details, also using ComBat batch correction) as fixed-effect factors, (2) an ASD-by-ADHD interaction term, and (3) linear and quadratic age, FSIQ, and a total brain measure (i.e., CT_0_ or SA_total_, respectively) as continuous covariates:

$$\begin{aligned} Y_{i} = & \beta_{0} + \beta_{1} {\text{ASD}} + \beta_{2} {\text{ADHD}} + \beta_{3} {\text{ASD}}*{\text{ADHD}} + \beta_{4} {\text{Sex}} + \beta_{5} {\text{Age}} + \beta_{6} {\text{Age}}^{2} \\ & + \beta_{7} {\text{FSIQ}} + \beta_{8} {\text{Site}} + \beta_{9} {\text{total}}\;{\text{Brain}} + \varepsilon_{i} \\ \end{aligned}$$

where *ε*_*i*_ is the residual error at vertex *i*. Continuous covariates were mean centered across groups to improve interpretability of the coefficients. Corrections for multiple comparisons across the whole brain were performed using random field theory (RFT) based cluster analysis with a cluster-forming and cluster-based threshold of *p* < 0.05 (two-tailed). Effect sizes associated with each model term were assessed using *Cohen’s f*, where values of 0.25, 0.5, and 0.75 indicate small, medium, and large effects, respectively.

To specifically identify neuroanatomical differences between ASD individuals with and without a co-occurring diagnosis of ADHD, we fitted an additional GLM on a subset of the total sample that only included participants with ASD and examined the main effect of ADHD (*N* = 312), i.e.,

$$Y_{i} = \beta_{0} + \beta_{1} {\text{Group}} + \beta_{2} {\text{Sex}} + \beta_{3} {\text{Age}} + \beta_{4} {\text{Age}}^{2} + \beta_{5} {\text{FSIQ}} + \beta_{6} {\text{Site}} + \beta_{7} {\text{total}}\;{\text{Brain}} + \varepsilon_{i }$$

### Gene expression decoding analysis

To link our neuroanatomical findings to putative genomic underpinnings, we leveraged the spatial gene expression data from the Allen Human Brain Atlas (AHBA; [27]) to identify a list of genes with a spatial pattern of expression that resembles the neuroanatomical patterns highlighted by our statistical neuroimaging analyses. To this aim, we initially uploaded the statistical t-maps associated with the main effect of ASD, the main effect of ADHD, as well as the ASD-by-ADHD interaction term for CT and SA (Fig. 4A, C) to the Neurovault server (https://neurovault.org). Next, using python code embedded within Neurovault and Neurosynth (https://neurosynth.org), we performed a gene expression decoding analysis that statistically assesses the spatial correlation between our statistical maps and the pattern of gene expression for each of a total of 20,787 protein coding genes [46]. To do so, the six AHBA donor brains are initially co-registered with the MNI atlas (also used by FreeSurfer) using nonlinear registration (transcriptomic alignment). At each sampling site (i.e., probe), a spherical region-of-interest (ROI) is drawn (default radius *r* = 4 mm), and the statistical test parameter in each FreeSurfer overlay was averaged within each ROI. This resulted in a spatial vector of values for each donor, which was subsequently correlated with the normalized gene expression data. Here, the analysis constructs a linear model for each donor brain, where the slopes encode the spatial correlation between each gene’s expression pattern to the statistical neuroimaging map (random effects model). In line with the input maps, these analyses were restricted to cortical tissue. The slopes are then subjected to a one-sample t test to identify genes whose spatial expression patterns are consistently highly similar to the imaging maps (i.e., across donor brains). The derived list of genes was thresholded at *p* < 0.01. We chose this ‘liberal’ threshold as this analysis did not constitute a hypothesis test per se, but rather a selection step aimed at yielding an initial list of genes for the subsequent enrichment analyses. Given that both sides of our imaging contrasts were of equal relevance, we considered both positive and negative t-statistic values.

In addition to the Neurosynth decoding approach, we performed gene expression decoding using a General Least Squares (GLS) approach that also accounted for spatial autocorrelations in embedded transcriptomic maps, to assess the robustness of our findings. The GLS approach is described in detail in Additional file 1: General Least Square (GLS)-decoding.

### Gene enrichment analyses

Next, we performed several gene enrichment analyses to establish the biological relevance and functional role of decoded genes. All enrichment testing was performed using the GeneOverlap package in R (https://doi.org/10.18129/B9.bioc.GeneOverlap). Specifically, we tested the decoded gene lists for an enrichment with different gene sets known to be associated with ASD at the genetic and transcriptomic level. At the genetic level, this included the 102 rare and de novo protein truncating variants identified in the largest exome sequencing study of autism worldwide [47]. We also included an ASD-related gene list compiled by SFARI (categories S, 1, 2, and 3 downloaded in November 2020 from https://gene.sfari.org/). At the transcriptomic level, we included a list of differentially expressed genes (DEGs) (upregulated/downregulated) in post-mortem cortex tissue in ASD [48], and genes that are differentially expressed in specific cell types in ASD [49]. Moreover, we included genes from differentially expressed co-expression modules in ASD that map onto specific biological processes [50]. ADHD-related genes were derived based on a GWAS study published by Demontis and colleagues [33]. Here, we used the MAGMA plug-in on the FUMA GWAS annotation platform (https://fuma.ctglab.nl) to perform a GWAS analysis [51]. Here, variants were mapped onto genes based on their exact position, and aggregated association *p* values were calculated for each gene. Taking into account the sample composition, a European ancestry reference from 1000 Genomes phase 3 was used as reference panel. Bonferroni correction was used to set the significance threshold (correcting for all *N* = 10,894 gene sets tested from MsigdB v5.2; [52]) The resulting gene list consisted of *N* = 22 ADHD-related genes, which was used for further gene enrichment analyses. Our enrichment tests generated enrichment odds ratios, hypergeometric *p* values, and FDR-corrected *p* values using a background total of the 20,787 Neurosynth genes. Last, we examined the percentage overlap between genes associated with both main effects and the interaction term relative to the total number of genes significantly associated with the imaging phenotypes (see Additional file 1: Figure S7 for details).

## Results

### Subject demographics

There was no significant difference in the ratio of males to females between any of the four subgroups (*p* = 0.06642) (Table 1). There was, however, a significant difference in age between the ASD group and the co-occurring group (ASD only: 18.45 ± 5.6; ASD + ADHD: 16.38 ± 5.30; *p* = 0.0061) (Table 1). Subgroups also differed significantly in FSIQ (*p* < 2e−16) [with TD having the highest IQ (108.44 ± 14.1) and the ADHD only group the lowest (79.8 ± 21.1)] (Table 1). We therefore covaried for these potential confounds in all subsequent analyses.

### Main effect of ASD and ADHD on CT and SA

**Fig. 1:**
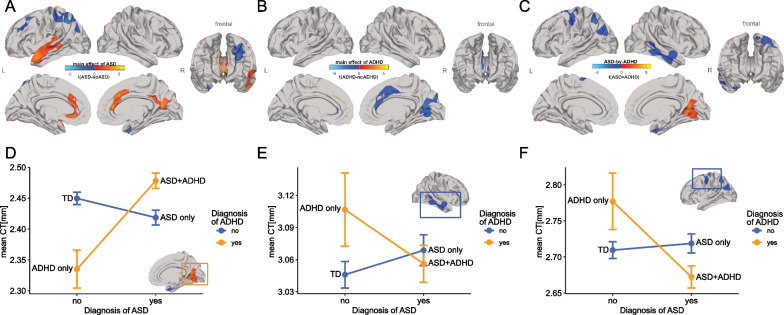
Differences of cortical thickness for main effects of ASD, ADHD, and ASD-by-ADHD interaction. **A**–**C** Random field theory (RFT)-based cluster corrected t-maps (*p* < 0.05, 2-tailed) for CT. **A** Significant decreases in CT in ASD compared to non-ASD are displayed in blue and significant increases are displayed in orange. **B** Significant decrease in CT in ADHD compared to non-ADHD is displayed in blue and significant increase is displayed in orange, **C** the ASD × ADHD interaction effect for CT, **D** mean CT at right cingulate cortex for the interaction effect, **E** mean CT at right temporal lobe for the interaction effect and **F** mean CT at left parietal cortex for the interaction effect. *ASD* autism spectrum disorder, *ADHD* attention-deficit/hyperactivity disorder, *TD* typically developing, *t* t-statistic, *CT* cortical thickness, *L* left, *R* right

**Fig. 2:**
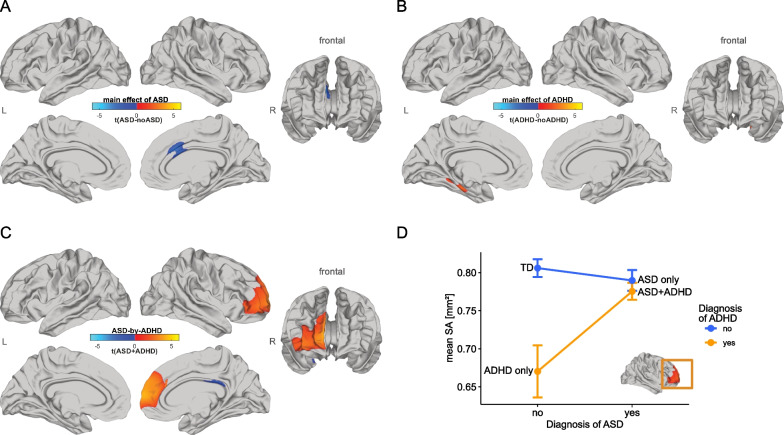
Differences of surface area for main effects of ASD, ADHD, and ASD-by-ADHD interaction. **A**–**C** Random field theory (RFT)-based cluster corrected t-maps (*p* < 0.05, 2-tailed) for SA. **A** Significant decrease in SA in ASD compared to non-ASD is displayed in blue and significant increase is displayed in orange. **B** Significant decrease in SA in ADHD compared to non-ADHD is displayed in blue and significant increase is displayed in orange, **C** the ASD × ADHD interaction effect for SA. **D** Mean SA at right frontal gyrus for the interaction effect. *ASD* autism spectrum disorder, *ADHD* attention-deficit/hyperactivity disorder, *TD* typically developing, *t* t-statistic, *SA* surface area, *L* left, *R* right

Following RFT-based cluster-correction (*p* < 0.05, two-tailed), the main effect of autism was associated with increased CT in the anterior-cingulate cortex (ACC; approximate Brodmann areas (BA) 24/33), in the left superior and middle temporal gyrus (BA 21/22), and the right precuneus cortex (BA 31). By contrast, autism was associated with decreased CT in the parietal cortex (BA 7) and the middle frontal gyrus (BA 6/8/9) (Fig. 1A). We also found that the main effect of autism was associated with decreased SA in the right anterior-cingulate cortex (BA 24/33) relative to non-autistic individuals (Fig. 2A; Additional file 1: Table S2). For the main effect of ADHD, we observed decreases in CT in right superior frontal gyrus (BA 6/8/9), right cingulate cortex (BA 23/24/33), and the right precuneus cortex (BA 31) (Fig. 1B). ADHD was also associated with increased SA in left parahippocampal gyrus (BA 27/28) only (Fig. 2B; Additional file 1: Table S3; see Additional file 1: Figure S3 for unthresholded t-maps). Vertex-level effect sizes (*Cohen’s f*) for the main effects of autism and ADHD, and all other model terms are displayed in the Additional file 1: Figure S4.

### Significant interactions between ASD and ADHD

In addition to the main effects of group, we examined an ASD-by-ADHD interaction term. We identified several cortical regions, where the neuroanatomy of autism (or ADHD) was significantly modulated by co-occurring ADHD (or ASD) including the left parietal cortex (BA 7), precentral and superior frontal gyrus (BA 6/8), the right temporal gyrus (BA 20/21/22), cingulate cortex (BA 23/24/33), and precuneus cortex (BA 31) for measures of CT (Fig. 1C). In these regions, we observed different types of interactions. For example, in the cingulate cortex CT in the ASD + ADHD group was significantly increased compared to the other groups (Fig. 1D), whereas in the precentral gyrus CT in the ASD + ADHD group was significantly decreased compared to all other groups (Fig. 1F). We also found a significant cluster in the right temporal gyrus with no significant difference between both ASD groups. Here, the interaction was mainly driven by the difference between individuals with ADHD and TD controls, which was significantly increased for measures of CT (Fig. 1E; see Additional file 1: Figure S5A for interaction plots of all significant clusters). For measures of SA, significant differences in the right dorsolateral prefrontal gyrus (BA 46) (Fig. 2C) were found. Here, the interaction was mainly driven by the difference between individuals with ADHD and TD controls, which was significantly decreased, while small or no differences were observed between controls and both autism subgroups (Fig. 2D; see Additional file 1: Figure S5B for interaction plots of all significant clusters; Additional file 1: Table S4).

### Significant differences between ASD + ADHD and ASD only

**Fig. 3:**
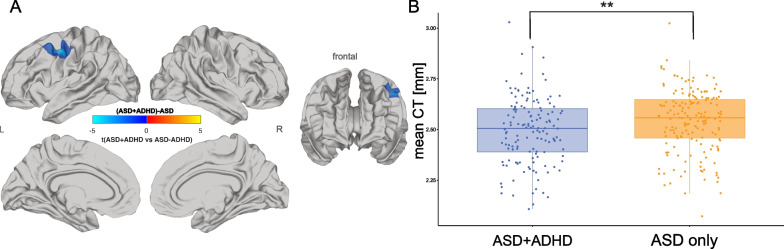
Differences of cortical thickness for ASD + ADHD versus ASD only. Random-field-theory-based cluster-corrected t-maps (*p* < 0.05, 2-tailed). **A** Significant decrease in CT in ASD + ADHD compared to ASD only is displayed in blue and significant increase is displayed in orange, **B** mean CT at left parietal cortex for the ASD + ADHD subgroup (blue) and the ASD only subgroup (yellow). *ASD* autism spectrum disorder, *ADHD* attention-deficit/hyperactivity disorder, *t* t-statistic, *CT* cortical thickness, *L* left, *R* right. **p < 0.01

To specifically identify differences between the ASD + ADHD and the ASD only group, we examined a subsample of individuals with ASD. We observed that individuals with ASD + ADHD had significantly decreased CT relative to individuals with ASD only in the left precentral, caudal middle frontal, and postcentral gyrus (BA 6/8/9) (Fig. 3; Additional file 1: Table S4). There were no significant differences for measures of SA.

### Gene set enrichment analyses

**Fig. 4:**
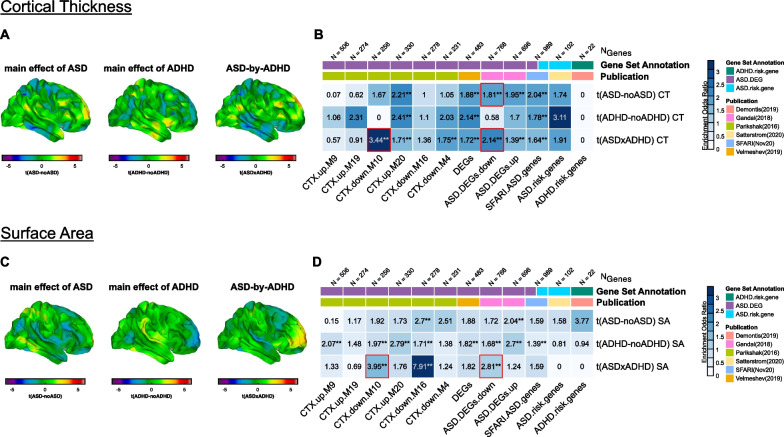
Genomic underpinnings of neurodevelopmental deviations in cortical thickness and surface area in ASD, ADHD, and ASD + ADHD. Panel **A** and **C** show the t-maps of the main effect of ASD, the main effect of ADHD and the ASD-by-ADHD interaction term for measures of CT (**A**) and SA (**C**). Panel **B** (CT) and **D** (SA) show significant odds ratios at a false discovery rate (FDR) corrected *p* threshold of 0.05 resulting from the gene set enrichment analyses for genes expressed in the different output maps. Gene sets were subdivided into sets with differential gene expression in ASD, sets representing ASD risk genes, and a set representing ADHD risk genes. Gene sets are annotated and labeled based on their original publication. *ASD* autism spectrum disorder, *ADHD* attention-deficit/hyperactivity-disorder, *CTX* cortex, *DEG* differentially expressed gene, *down* downregulated expression in ASD, *up* upregulated expression in ASD, *NGenes* number of genes in each gene set, *CT* cortical thickness, *SA* surface area. *p < 0.05 (FDR-corrected), **p < 0.01 (FDR-corrected); red squares indicate consistently significant enrichments across both approaches (see Additional file11: Figure S6)

Here, we decoded the t-maps for the main effects of autism and ADHD, as well as the CT and SA maps for the ASD-by-ADHD interaction terms (i.e., six maps in total). This resulted in sets of (1) *N*_CT_ = 598 and *N*_SA_ = 259 significant genes associated with the main effect of autism for CT and SA, respectively, (2) *N*_CT_ = 272 and *N*_SA_ = 1005 significant genes associated with the main effect of ADHD, and (3) *N*_CT_ = 1258 and *N*_SA_ = 233 significant genes associated with the ASD-by-ADHD interaction term. Within these gene sets, we observed a significant enrichment of genes known to be associated with autism, and particularly for genes that are known to be differentially expressed in autism during childhood and adolescence (see Fig. 4 for details). More specifically, in all decoded CT maps (i.e., for main effect of autism, ADHD, and the interaction term), we observed a significant enrichment of gene co-expression module CTX.M20, which is known to be upregulated in the autism cortex and has been linked to Gene Ontology terms representing developmental processes and the regulation of cell differentiation [50]. Moreover, we observed a significant enrichment of autism genes listed in the SFARI database. Notably, there was no significant enrichment of autism- or ADHD-related risk genes resulting from GWAS studies. However, these were also among the smallest gene sets tested with only *N* = 22 genes for ADHD (Fig. 4A, B).

Different gene sets were found to be enriched in the SA maps (Fig. 4C, D). Here, in all decoded maps, we observed an enrichment of gene co-expression module CTX.M16, which is associated with neuronal markers and synaptic genes [53], and is known to be downregulated in the autism cortex. In contrast to measures of CT, however, where most gene sets were enriched in both main effects for autism and ADHD, gene set enrichments were more prominent in the main effect of ADHD for measures of SA. Autism-related genes hence seem to have a pattern of expression that more closely resembles the differences in CT in autism rather than differences in SA. Moreover, autism-related genes are equally important to the neuroanatomy underpinning ADHD symptomatology, particularly when examining measures of SA. Gene sets resulting from the GLS-decoding approach are shown in the Additional file 1: Figure S6.

Overall (i.e., across gene sets), there was little overlap between genes significantly associated with the main effect of ASD, ADHD, and/or the ASD-by-ADHD interaction terms on the transcriptomic level (see Additional file 1: Figure S7). More specifically, a total of 1,3% of all genes identified as being significantly enriched in any of the imaging phenotypes was shared between the main effect of ASD and ADHD for measures of CT, and a total of 8.1% of genes associated with these main effects for measures of SA.

## Discussion

This study examined neuroanatomical differences between autistic individuals with and without co-occurring ADHD relative to individuals with ADHD only and non-autistic controls. Moreover, to bridge the gap between macroscopic and microscopic differences, we examined whether the spatial patterns of neuroanatomical differences in CT and SA are enriched for genes implemented in the etiology of autism and ADHD, and genes known to be differentially expressed in autism. We established that it is possible to separate the effect of autism from the effect of ADHD on the level of neuroanatomy based on measures of CT and SA. Notably, we also observed significant ASD-by-ADHD interactions, and significant differences in CT between ASD individuals with ADHD and those without. This suggests that the neuroanatomy of ASD is significantly modulated by co-occurring ADHD. Further we showed a significant association of autism and ADHD-related patterns of neuroanatomical variability with autism—but not ADHD-related genes. Our study thus provides important novel insights into the neurobiological and putative genomics underpinning the complex clinical phenotype of autism.

### Main effect of ASD and ADHD on CT and SA

Neuroanatomical differences in autism and ADHD are well documented and primarily affect fronto-temporal and fronto-parietal regions for autism [2], as well as cingulate cortices for ADHD [11]. In the present study, we examined the neuroanatomical underpinnings of autism and ADHD within a 2 × 2 factorial design that included (1) ASD individuals with co-occurring ADHD, (2) ASD only individuals, (3) ADHD only individuals, and (4) non-autistic controls. While it remains a topic of debate whether autism should be encoded as a categorical variable [54], this design allowed us to identify a set of brain regions where neuroanatomical variability in CT and SA are uniquely attributable to either autism or ADHD. By examining both main effects, we were able to separate autism from ADHD based on patterns of neuroanatomical variability in fronto-temporal regions (e.g., left superior and middle temporal gyrus), and in limbic and prefrontal regions (i.e., cingulate cortex, parahippocampal gyrus, and the superior frontal gyrus). Many of the brain regions associated with the main effect of ASD have previously been linked to the wider neural systems that mediate functions related to social cognition and/or emotional processing, i.e., core autism traits [9]. Prefrontal areas such as the superior frontal gyrus, which showed significant differences in CT for the main effect of ADHD, are important for executive functioning, attention and motor planning, which are all functions known to be impaired in ADHD [55, 56]. Our findings of significant neuroanatomical differences associated with ASD and ADHD are thus in line with previous reports using a categorical approach; however, it does not rule out the necessity of future studies using dimensional approaches. While a categorical approach is particularly well suited to examine neuroanatomical differences associated with both ASD and ADHD (as well as their interaction), future dimensional research would complement our study by accounting for the inter-individual differences in the broader phenotypes of ASD and ADHD. To date, autism diagnostic is still based on a categorical cutoff that remains largely unchanged across the human lifespan.

In addition, as studies examining the neuroanatomical underpinnings of autism and co-morbid ADHD are still rare, and there is currently little knowledge on basic case–control differences, elicited in adequately powered samples. In terms of ASD, several lines of ASD research also converge in suggesting that the condition might be most effectively understood as a categorical fixed effect rather than a dimensional construct (for review see [57]). For example, Frazier et al. [58] examined several indicators of ASD (e.g., eye gaze metrics from social stimulus paradigms), establishing a categorical data structure that closely corresponded to a diagnosis of ASD across measures [58]. ADHD, on the other hand, is widely considered a dimensional construct with fluctuations in symptom severity and profiles across the human lifespan [11, 59, 60]. For example, only 15% of children with ADHD still show criteria for a diagnosis during adulthood [60], and symptoms often also change from a more externalizing nature during childhood to a more internalizing nature during adulthood [11, 59]. Future studies might therefore benefit from employing both a categorical and a dimensional approach to examine the neuroanatomy associated with ASD and ADHD.

The effect sizes associated with the main effect of ASD and ADHD were relatively small, overall, and did not exceed a value of 0.2 on the vertex level. Small-to-medium effects associated with between-group differences in neuroanatomy have previously been observed even in large-scale neuroimaging studies with *N* > 500 that compare ASD or ADHD individuals with neurotypical controls [12, 14]. The low effects reported by neuroimaging studies seem to be driven by small differences in mean, and larger phenotypic variability in neurodiverse study population [44], thus highlighting the significant inter-individual heterogeneity that is characteristic for neurodevelopmental conditions on the level of etiology, neurobiology, and symptomatology. Taken together, our findings indicate that autism and ADHD may have separable neuroanatomical underpinnings that may mediate differences in clinical phenotypes. The main effects of autism and ADHD are, however, only interpretable in the absence of a significant ASD-by-ADHD interaction.

### Significant interactions between ASD and ADHD

While previous studies have focused on the effects of ASD and ADHD in separate case–control designs [14, 61], we examined to what extent, and where in the brain, the neuroanatomy of ASD is modulated by ADHD symptomatology (i.e., the ASD-by-ADHD interaction). We observed a significant ASD-by-ADHD interaction term in the right temporal gyrus, the right cingulate gyrus and the left precentral gyrus for measures of CT, and in the right dorsolateral prefrontal gyrus for measures of SA. Some of these clusters (right temporal gyrus and right dorsolateral prefrontal gyrus) were mainly driven by neuroanatomical differences between individuals with ADHD only relative to controls, while small or no differences were observed between non-autistic controls and autistic individuals (including those with co-occurring ADHD). ADHD-related neuroanatomical variability in these brain regions thus seems to be modulated, or even masked, by also having a diagnosis of autism. However, other clusters associated with the ASD-by-ADHD interaction term, e.g., in the right cingulate gyrus and left precentral gyrus, also showed significant differences between the ASD participants with co-occurring ADHD and those with ASD only. A significant interaction between autism and ADHD indicates that the neuroanatomical underpinnings of ADHD in autistic individuals cannot be explained by either a diagnosis of autism or a diagnosis of ADHD alone.

### Significant differences between ASD + ADHD and ASD only

Our additional analysis within the ASD ± ADHD subsample showed significant differences between both groups in the left precentral gyrus for measures of CT. Here, individuals with co-occurring autism and ADHD had a significantly thinner cortex compared to those individuals with autism alone. For measures of SA, no significant clusters for this contrast were observed. Taken together, our findings suggest that (1) the neuroanatomy of autism is significantly modulated by co-occurring ADHD symptomatology, and (2) although ADHD is highly co-occurring in autism, both conditions seem to have separable neuroanatomical underpinnings.

### Gene set enrichment analyses

In a next analysis step, we examined whether the patterns of CT and SA attributed to the main effects and the interaction term are linked to the genetic etiology of autism or ADHD. Similar to Romero-Garcia et al. [62], we found that all decoded CT maps were enriched for genes and co-expression modules that have previously been implicated in the etiology of autism, and particularly for genes associated with brain development and the regulation of cell differentiation [50]. For measures of SA, an enrichment of genes and co-expression modules that are associated with neuronal markers and synaptic genes was found [53]. Notably, the pattern of neuroanatomical differences in SA associated with the main effect of ADHD also showed a robust enrichment of autism-related genes, implying that these genes may not be specific to autism, but may also affect the neuroanatomy of other neurodevelopmental conditions such as ADHD.

This finding agrees with previous genetic studies demonstrating that ASD and ADHD are genetically correlated, with a SNP-based genetic correlation of 0.37 [34]. Many of the genetic loci implicated in ASD and ADHD are therefore ‘pleiotropic’, i.e., can influence two or more clinical phenotypes. Moreover, the effects of ASD susceptibility genes on the brain are known to be pleiotropic and may exert their influence via gene regulatory mechanisms during childhood and adolescence [30, 63, 64]. This could also account for highly individualized patterns of neuroanatomical variations or ‘fingerprints’ that are commonly observed in ASD [44]. It is therefore possible that similar genotypes underpin distinct phenotypes, which could explain why we observed an enrichment of ASD-related genes both in the ASD and ADHD imaging phenotype, even though the neuroanatomical patterns characteristic for each phenotype were different.

Moreover, while ASD and ADHD are known to be genetically related, little is currently known about the functional involvement of genes that are specific to ASD and/or ADHD. Moreover, distinct imaging phenotypes could be caused by more complex genetic interactions between ASD- and ADHD-related genes, which cannot be identified based on the gene set enrichment analyses performed in the present study. This highlights the need for conducting future large-scale genome wide association studies (GWAS) to link genetic variation associated with neurodevelopmental conditions to differences in neuroanatomical phenotypes.

Also, while we observed a significant enrichment of autism-related genes, there was no significant enrichment of ADHD risk genes in the main effect of ASD or ADHD. However, the number of recognized ADHD-susceptibility genes remains as yet small, which limits the statistical power of enrichment tests. Moreover, little is currently known about the functional role of ADHD genes and their impact on cortical gene expression. Future genetic and/or transcriptomic studies are therefore needed to provide further insights into the genomic underpinnings of ADHD, and to expand the set of genes that might be utilized to link ADHD-related differences in brain anatomy to putative underlying mechanisms.

### Limitations

The present study needs to be interpreted in light of several limitations. Although we employed a multicenter design to overcome single-site recruitment limitations, our sample size of *N* = 25 individuals in the ADHD only group is relatively small compared to the other subgroups, which limits the generalizability of the results. Also, we assigned subjects into ADHD positive groupings according to the DSM-5 rating scale. While this is sufficient for obtaining a research diagnosis of ADHD, our approach did not take into account for how long the symptoms have been present, which is typically considered when assessing ADHD in the clinical setting. It will therefore be important to repeat our analyses in a bigger sample of pure ADHD cohorts that were diagnosed according to clinical gold standards to make our study comparable to previous findings in these cohorts. Furthermore, future studies employing a dimensional approach are also required to provide a more nuanced picture of the heterogeneity associated with autism. Last, our gene expression decoding analysis was based on the Allen Human Brain Atlas [27], which is the most comprehensive gene expression atlas to date. However, the Allen atlas is based on adult donors exclusively, and provides a coverage that is significantly lower than the spatial resolution of our neuroimaging data. We therefore acknowledge the importance of repeating the analyses in high-resolution age-specific gene expression data sets once these become available, to corroborate the important link between molecular and macroscopic pathology in autism.

## Conclusion

Our findings indicate that the neuroanatomy of ASD is significantly modulated by ADHD, and that autistic individuals with co-occurring ADHD may have specific neuroanatomical underpinnings potentially mediated by atypical gene expression.

### Supplementary Information

**Additional file 1**. **Methods. 1.** Sample Description. **2.** MRI Data Quality Assessments. **3.** Effects of Intellectual Disability. **4.** Site effects. **5.** General Least Square (GLS)-decoding accounting for autocorrelations in spatially embedded transcriptomic maps. **Figure S1A.** Effects of Intellectual Disability (ID) for measures of cortical thickness (CT). **Figure S1B.** Effects of Intellectual Disability (ID) for measures of cortical thickness (CT). **Figure S1C.** Effects of Intellectual Disability (ID) for measures of surface area (SA). **Figure S1D.** Effects of Intellectual Disability (ID) for measures of surface area (SA). **Figure S2A.** Effects of acquisition sites for measures of cortical thickness (CT). **Figure S2B.** Effects of acquisition sites for measures of cortical thickness (CT). **Figure S2C.** Effects of acquisition sites for measures of surface area (SA). **Figure S2D.** Effects of acquisition sites for measures of surface area (SA). **Figure S3.** Significant differences of cortical thickness (CT) and surface area (SA) for the main effects of ASD, ADHD and the ASD-by-ADHD interaction term. **Figure S4.** Effect size plots for individual model terms. **Figure S5A.** Interaction plots for all significant clusters for measures of cortical thickness (CT). **Figure S5B.** Interaction plots for all significant clusters for measures of surface area (SA). **Figure S6.** Gene enrichments as resulting from the GLS-decoding approach **Figure S7.** Overlap between significant decoded genes. **Table S1.** MRI Acquisition Parameters across sites. **Table S2.** Vertex-wise differences in CT and SA for the main effect of ASD. **Table S3.** Vertex-wise differences in CT and SA for the main effect of ADHD. **Table S4.** Vertex-wise differences in CT and SA for the ASD-by-ADHD interaction term. **Table S5.** Vertex-wise differences in CT for the main effect of ASD + ADHD.

## Data Availability

Due to ethical restrictions, we are currently not able to make the data publicly available. However, it is planned to release the data for perusal of the wider scientific community within the next couple of years once legal requirements are in place.

## Abbreviations

ASD: Autism spectrum disorders
ADHD: Attention-deficit/hyperactivity disorder
GM: Gray matter
CT: Cortical thickness
LEAP: Longitudinal European Project
SA: Surface area
AHBA: Allen Human Brain Atlas
SCZ: Schizophrenia
GWAS: Genome-wide association studies
SNP: Single nucleotide polymorphisms
TD: Typically developing
GLM: General linear model
FSIQ: Full-scale IQ
ID: Intellectual disability
RFT: Random field theory
BA: Brodmann areas
