# Supplementary material for: The neuroanatomical substrates of autism and ADHD and their link to putative genomic underpinnings
Source: Mol Autism. 2023 Oct 4;14:36. doi: 10.1186/s13229-023-00568-z (PMC10552404; doi:10.1186/s13229-023-00568-z)
Supplement: Supplementary file 1 — Additional file 1. Methods. 1. Sample Description. 2. MRI Data Quality Assessments. 3. Effects of Intellectual Disability. 4. Site effects. 5. General Least Square (GLS)-decoding accounting for autocorrelations in spatially embedded transcriptomic maps. Figure S1A. Effects of Intellectual Disability (ID) for measures of cortical thickness (CT). Figure S1B. Effects of Intellectual Disability (ID) for measures of cortical thickness (CT). Figure S1C. Effects of Intellectual Disability (ID) for measures of surface area (SA). Figure S1D. Effects of Intellectual Disability (ID) for measures of surface area (SA). Figure S2A. Effects of acquisition sites for measures of cortical thickness (CT). Figure S2B. Effects of acquisition sites for measures of cortical thickness (CT). Figure S2C. Effects of acquisition sites for measures of surface area (SA). Figure S2D. Effects of acquisition sites for measures of surface area (SA). Figure S3. Significant differences of cortical thickness (CT) and surface area (SA) for the main effects of ASD, ADHD and the ASD-by-ADHD interaction term. Figure S4. Effect size plots for individual model terms. Figure S5A. Interaction plots for all significant clusters for measures of cortical thickness (CT). Figure S5B. Interaction plots for all significant clusters for measures of surface area (SA). Figure S6. Gene enrichments as resulting from the GLS-decoding approach Figure S7. Overlap between significant decoded genes. Table S1. MRI Acquisition Parameters across sites. Table S2. Vertex-wise differences in CT and SA for the main effect of ASD. Table S3. Vertex-wise differences in CT and SA for the main effect of ADHD. Table S4. Vertex-wise differences in CT and SA for the ASD-by-ADHD interaction term. Table S5. Vertex-wise differences in CT for the main effect of ASD + ADHD. [file 13229_2023_568_MOESM1_ESM.docx]

**Supplementary information**

Table of Contents

[Supplementary Methods 2](#_Toc143865396)

[1. Sample Description 2](#_Toc143865397)

[2. MRI Data Quality Assessments 2](#_Toc143865398)

[3. Effects of Intellectual Disability 3](#_Toc143865399)

[4. Site effects 3](#_Toc143865400)

[5. General Least Square (GLS)-decoding accounting for autocorrelations in spatially embedded transcriptomic maps 4](#_Toc143865401)

[Supplementary Figures 6](#_Toc143865402)

[Figure S1A: Effects of Intellectual Disability (ID) for measures of cortical thickness (CT). 6](#_Toc143865403)

[Figure S1B: Effects of Intellectual Disability (ID) for measures of cortical thickness (CT). 7](#_Toc143865404)

[Figure S1C: Effects of Intellectual Disability (ID) for measures of surface area (SA). 8](#_Toc143865405)

[Figure S1D: Effects of Intellectual Disability (ID) for measures of surface area (SA). 9](#_Toc143865406)

[Figure S2A: Effects of acquisition sites for measures of cortical thickness (CT). 10](#_Toc143865407)

[Figure S2B: Effects of acquisition sites for measures of cortical thickness (CT). 11](#_Toc143865408)

[Figure S2C: Effects of acquisition sites for measures of surface area (SA). 12](#_Toc143865409)

[Figure S2D: Effects of acquisition sites for measures of surface area (SA). 13](#_Toc143865410)

[Figure S3: Significant differences of cortical thickness (CT) and surface area (SA) for the main effects of ASD, ADHD and the ASD-by-ADHD interaction term. 14](#_Toc143865411)

[Figure S4: Effect size plots for individual model terms. 15](#_Toc143865412)

[Figure S5A: Interaction plots for all significant clusters for measures of cortical thickness (CT). 16](#_Toc143865413)

[Figure S5B: Interaction plots for all significant clusters for measures of surface area (SA). 17](#_Toc143865414)

[Figure S6: Gene enrichments as resulting from the GLS-decoding approach. 18](#_Toc143865415)

[Figure S7: Overlap between significant decoded genes. 19](#_Toc143865416)

[Supplementary Tables 20](#_Toc143865417)

[Table S1:MRI Acquisition Parameters across sites 20](#_Toc143865418)

[Table S2: Vertex-wise differences in CT and SA for the main effect of ASD 20](#_Toc143865419)

[Table S3: Vertex-wise differences in CT and SA for the main effect of ADHD 21](#_Toc143865420)

[Table S4: Vertex-wise differences in CT and SA for the ASD-by-ADHD interaction term 22](#_Toc143865421)

[Table S5: Vertex-wise differences in CT for the main effect of ASD+ADHD 22](#_Toc143865422)

[References 23](#_Toc143865423)

[The EU-AIMS LEAP consortium: 23](#_Toc143865424)

# Supplementary Methods

## Sample Description

All autism participants had a clinical diagnosis of autism according to DSM-5 or ICD-10 criteria. Further we assessed autism traits with the Autism Diagnostic Observation Schedule (ADOS) (1), and the Autism Diagnostic Interview-Revised (ADI-R) (2). Individuals with a clinical diagnosis who did not meet the cut-offs for autism, however, were not excluded to sample a broader autism phenotype in the general population. ﻿Exclusion criteria included a history of alcohol and/or substance abuse or dependence in the past, MRI contraindications as well as significant hearing or visual impairments. We further excluded all participants with no information on their DSM-5 ADHD rating (3).

## MRI Data Quality Assessments

Initially structural MRI data was available for a total of 708 individuals in the LEAP sample, which was acquired across six European sites: (i) Institute of Psychiatry, Psychology and Neuroscience, King’s College London (IoPPN/KCL, United Kingdom), (ii) Autism Research Centre, University of Cambridge (UCAM, United Kingdom), (ii) University Medical Centre Utrecht (UMCU, Netherlands), (iv) Radboud University Nijmegen Medical Centre (RUNMC, Netherlands), (v) Central Institute of Mental Health (CIMH, Germany), and (vi) the University Campus Bio-Medico (UCBM) in Rome, Italy. All available data was preprocessed using the default pipeline implemented in the FreeSurfer v6.0.0 software (<http://surfer.nmr.mgh.harvard.edu/>). The resulting surface reconstructions were assessed for reconstruction errors and rated by three independent raters. Following manual editing and re-preprocessing of the rated reconstructions a final sample of 638 individuals (n=359 with ASD, n=279 TD controls) was available.

## Effects of Intellectual Disability

A number of individuals within our sample had a mild intellectual disability (ID) defined as a full-scale IQ < 70 (TD: N=5, ASD: N=19, ADHD: N=12, ASD+ADHD: N=29). To further investigate the potential effects of ID, we analyzed a subset of our sample excluding participants with mild ID (N=65). This resulted in a total sample of N=468 participants (TD: N=191, ASD: N=151, ADHD: N=13, ASD+ADHD: N=113). After excluding ID individuals, groups still significantly differed in full-scale IQ (FSIQ) (p=7.26e-05, df=3, F=7.423), which is why we also covaried for FSIQ in the subset analyses. We established that for both CT and SA, the neuroanatomical patterns observed across subsamples (i.e., with and without ID participants) were comparable in terms of the overall pattern of t-test statistics (see Figure S1A & S1C). The t-maps associated with the fixed effects (main and interaction terms) were also highly spatially correlated between subsample analyses (Pearson’s r ranging between 0.651 and 0.738, Figures S1A & S1C). However, reducing the sample size by excluding mind ID individuals also meant that some clusters reaching statistical significance in the full sample, were no longer significant in the sample subset (Figures 1B & 1D).

## Site effects

As our study was a multi-center MRI investigation with parallel recruitment at six European sites, it was important to account for scanner- or site-related confounds that may be unrelated to the main effects and the interaction term, but may still have an impact on our findings. Site effects are typically accounted for via the inclusion of site as a fixed effect factor within the GLM, which makes it possible to explicitly model site effects at each cerebral vertex-level. However, a variety of other approaches accounting for site effects have been suggested in the literature, one of them being a ComBat batch adjustment method prior to the statistical analysis (4). Here, we therefore also examined the robustness of our results across different approaches. We find that there was little difference between the GLM and ComBat correction of site effects overall. (see Figure S2A & S2C). Applying ComBat correlation prior to statistical modelling did not significantly affect the spatially distribution of the t-test statistic overall, and there was a significant spatial correlation between t-maps across approaches (Pearson’s r ranging between 0.767 and 0.995). However, there were some differences on the cluster level, which are shown in Figure S2B and S2D for measures of CT and SA respectively. To make our findings comparable to previous publications based on the same sample (e.g. (5)), the findings of the GLM covarying for site effects as a fixed effect are shown in the main manuscript (see Figures 1 & 2, as well as Figure S3).

## General Least Square (GLS)-decoding accounting for autocorrelations in spatially embedded transcriptomic maps

To assess for the robustness of the findings resulting from the gene expression decoding in and enrichment analyses, we compared the results of the Neurosynth decoding approach to an alternative decoding technique that also accounted for autocorrelations in spatially embedded transcriptomic/neuroimaging maps (see Fulcher et al. for details (6)). Here, the data provided by the AHBA was initially pre-processed with the *abagen* toolbox (version 0.1.1; https://github.com/rmarkello/abagen), using the FreeSurfer fsaverage6 as standard space template (see (7) for details). This resulted in a sample-by-gene matrix of normalized mRNA expression values for each of 15,633 genes across 1,670 samples in cortical brain tissue. Samples were then allocated to fsaverage6 surface vertices via mesh representations of the six donor brains (8). At each sample vertex, a geodesic circle with 5mm radius was drawn, and the values in each FreeSurfer overlay were averaged within the vertex neighborhood. This resulted in a spatial vector of 1,670 elements for each imaging pattern, which was subsequently correlated with the normalized gene expression data. To identify genes significantly correlated with the imaging phenotype, a Generalized Least Squares (GLS) model accounting for spatial autocorrelations (α) was fitted for each gene. This model predicted the spatial distribution of the imaging data by the mRNA profile of each gene, covarying for a Gaussian autoregressive spatial correlation structure defined by vertex x,y,z coordinates, and donor as grouping factor. All models were fitted using the R package ‘nlme’ (version 3.1.153). Gene-level *p-*values were obtained based on the main effect of mRNA expression profile. Similar to the Neurosynth decoding approach, we retained genes with p < 0.01 for further analyses.

Based on the list of GLS-decoded genes, we repeated our enrichment analyses as described in the main body of the manuscript. The results of the gene enrichment analyses across GLS-decoded genes are shown in Figure S6. Here, gene sets that are consistently enriched across the Neurosynth and GLS-decoding approached are marked in red.

# Supplementary Figures

Figure S1A: Effects of Intellectual Disability (ID) for measures of cortical thickness (CT). *Spatial distribution of t-values associated with different model terms for measures of CT. Left panel: Test statistic for the main effect of ASD, ADHD, and the interaction terms based on the analysis within the full sample (i.e., including those with mild ID). Right panel: Test statistic associated with model terms based on the analysis within a smaller subset of the sample that excluded individuals with mild ID. Note: ASD: autism spectrum disorder; ADHD: attention-deficit/hyperactivity disorder; ID: intellectual disability; t: t-test statistic; r: Person’s r; p: p-value of Pearson’s correlation.*

Figure S1B: Effects of Intellectual Disability (ID) for measures of cortical thickness (CT). *(A-C) Random field theory (RFT)-based cluster corrected t–maps (p < 0.05, 2-tailed) for subgroup analysis. (A) Significant decreases in CT in ASD compared to non ASD are displayed in blue and significant increases are displayed in orange (B) Significant decrease in CT in ADHD compared to non ADHD is displayed in blue and significant increase is displayed in orange (C) the ASD×ADHD interaction effect for CT. Left panel: Significant clusters for the main effect of ASD, ADHD, and the interaction terms based on the analysis within the full sample (i.e., including those with mild ID). Right panel: Significant clusters associated with model terms based on the analysis within a smaller subset of the sample that excluded individuals with mild ID. Note: ASD: autism spectrum disorder; ADHD: attention-deficit/hyperactivity disorder; ID: intellectual disability;* t: t-statistic*.*

Figure S1C: Effects of Intellectual Disability (ID) for measures of surface area (SA). *Spatial distribution of t-values associated with different model terms for measures of SA. Left panel: Test statistic for the main effect of ASD, ADHD, and the interaction terms based on the analysis within the full sample (i.e., including those with mild ID). Right panel: Test statistic associated with model terms based on the analysis within a smaller subset of the sample that excluded individuals with mild ID. Note: ASD: autism spectrum disorder; ADHD: attention-deficit/hyperactivity disorder; ID: intellectual disability; t: t-test statistic; r: Person’s r; p: p-value of Pearson’s correlation.*

Figure S1D: Effects of Intellectual Disability (ID) for measures of surface area (SA). *(A-C) Random field theory (RFT)-based cluster corrected t–maps (p < 0.05, 2-tailed) for subgroup analysis. (A) Significant decreases in SA in ASD compared to non ASD are displayed in blue and significant increases are displayed in orange (B) Significant decrease in SA in ADHD compared to non ADHD is displayed in blue and significant increase is displayed in orange (C) the ASD×ADHD interaction effect for SA. Left panel: Significant clusters for the main effect of ASD, ADHD, and the interaction terms based on the analysis within the full sample (i.e., including those with mild ID). Right panel: Significant clusters associated with model terms based on the analysis within a smaller subset of the sample that excluded individuals with mild ID. Note: ASD: autism spectrum disorder; ADHD: attention-deficit/hyperactivity disorder; ID: intellectual disability;* t: t-statistic*.*

Figure S2A: Effects of acquisition sites for measures of cortical thickness (CT). *Spatial distribution of t-values associated with different model terms for measures of CT. Left panel: Test statistic for the main effect of ASD, ADHD, and the interaction terms based on the analysis treating site as a fixed effect variable. Right panel: Test statistic associated with model terms based on the analysis treating site as a random effect (i.e., using ComBat correction). Note: ASD: autism spectrum disorder; ADHD: attention-deficit/hyperactivity disorder; t: t-statistic; r: Person’s r; p: p-value of Pearson’s correlation.*

Figure S2B: Effects of acquisition sites for measures of cortical thickness (CT). *(A-C) Random field theory (RFT)-based cluster corrected t–maps (p < 0.05, 2-tailed) associated with different model terms for measures of CT. (A) Significant decreases in CT in ASD compared to non ASD are displayed in blue and significant increases are displayed in orange (B) Significant decrease in CT in ADHD compared to non ADHD is displayed in blue and significant increase is displayed in orange (C) the ASD×ADHD interaction effect for CT. Left panel: Significant clusters for the main effect of ASD, ADHD, and the interaction terms based on the analysis treating site as a fixed effect variable. Right panel: Significant clusters associated with model terms based on the analysis treating site as a random effect (i.e., using ComBat correction). Note: ASD: autism spectrum disorder; ADHD: attention-deficit/hyperactivity disorder; t: t-statistic.*

Figure S2C: Effects of acquisition sites for measures of surface area (SA). *Spatial distribution of t-values associated with different model terms for measures of SA. Left panel: Test statistic for the main effect of ASD, ADHD, and the interaction terms based on the analysis treating site as a fixed effect variable. Right panel: Test statistic associated with model terms based on the analysis treating site as a random effect (i.e., using ComBat correction). Note: ASD: autism spectrum disorder; ADHD: attention-deficit/hyperactivity disorder; t: t-statistic; r: Person’s r; p: p-value of Pearson’s correlation.*

Figure S2D: Effects of acquisition sites for measures of surface area (SA). *(A-C) Random field theory (RFT)-based cluster corrected t–maps (p < 0.05, 2-tailed) associated with different model terms for measures of SA. (A) Significant decreases in SA in ASD compared to non ASD are displayed in blue and significant increases are displayed in orange (B) Significant decrease in SA in ADHD compared to non ADHD is displayed in blue and significant increase is displayed in orange (C) the ASD×ADHD interaction effect for SA. Left panel: Significant clusters for the main effect of ASD, ADHD, and the interaction terms based on the analysis treating site as a fixed effect variable. Right panel: Significant clusters associated with model terms based on the analysis treating site as a random effect (i.e., using ComBat correction). Note: ASD: autism spectrum disorder; ADHD: attention-deficit/hyperactivity disorder; t: t-statistic.*


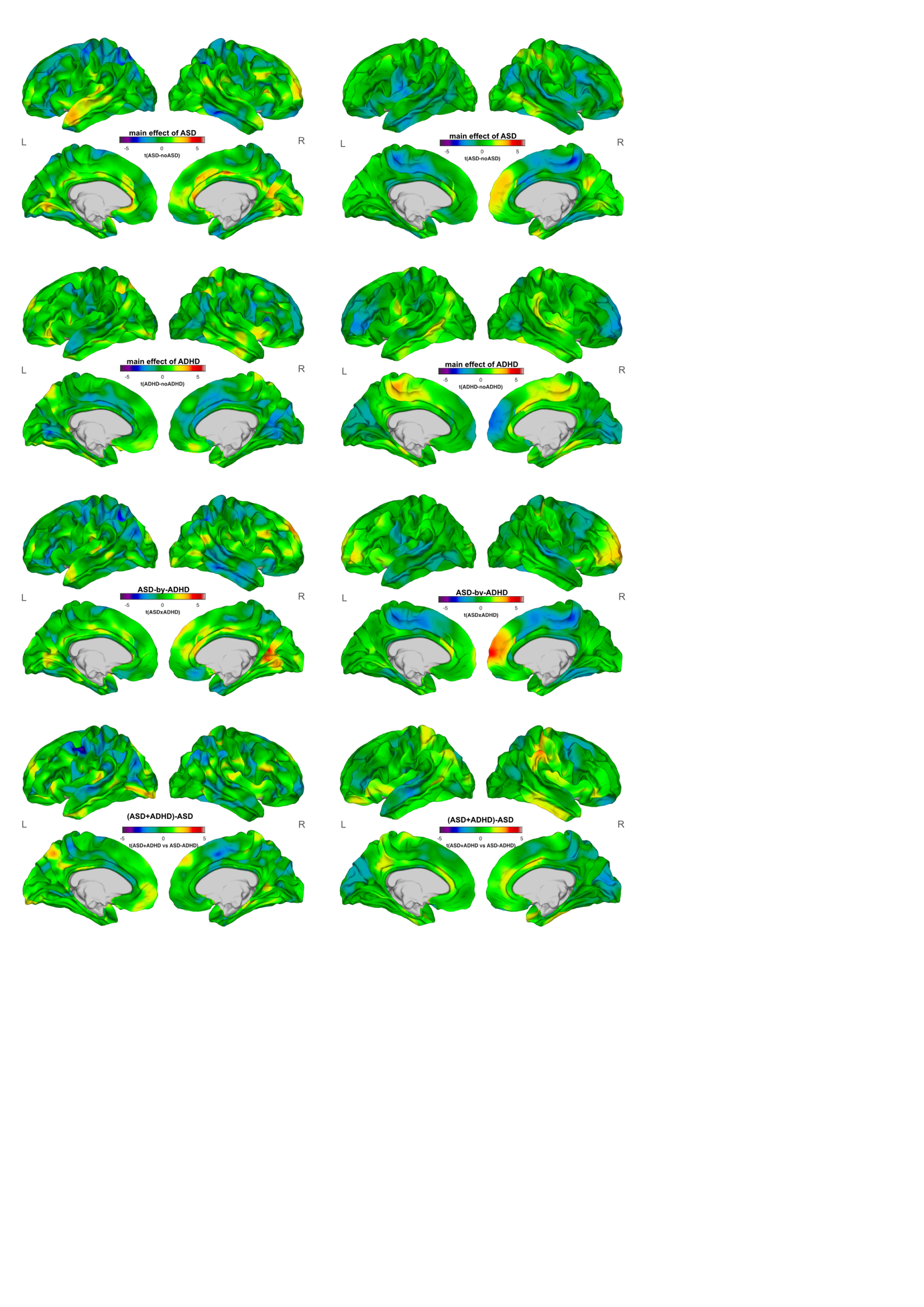


Figure S3: Significant differences of cortical thickness (CT) and surface area (SA) for the main effects of ASD, ADHD and the ASD-by-ADHD interaction term. *Left panel: Spatial distribution of t-values associated with different model terms for measures of CT. Right panel: Spatial distribution of t-values associated with different model terms for measures of SA. Note: L: left; R: right; ASD: autism spectrum disorder; ADHD: attention-deficit/hyperactivity disorder**.*

Figure S4: Effect size plots for individual model terms. Effect Sizes (Cohen’s f) for all components of the general linear model. Distribution of effects sizes across all vertices on the cortical surface for (A) CT and (B) SA. Note: ASD: autism spectrum condition; ADHD: attention deficit hyperactivity disorder; FSIQ: full-scale IQ; CT: cortical thickness; SA: surface area.


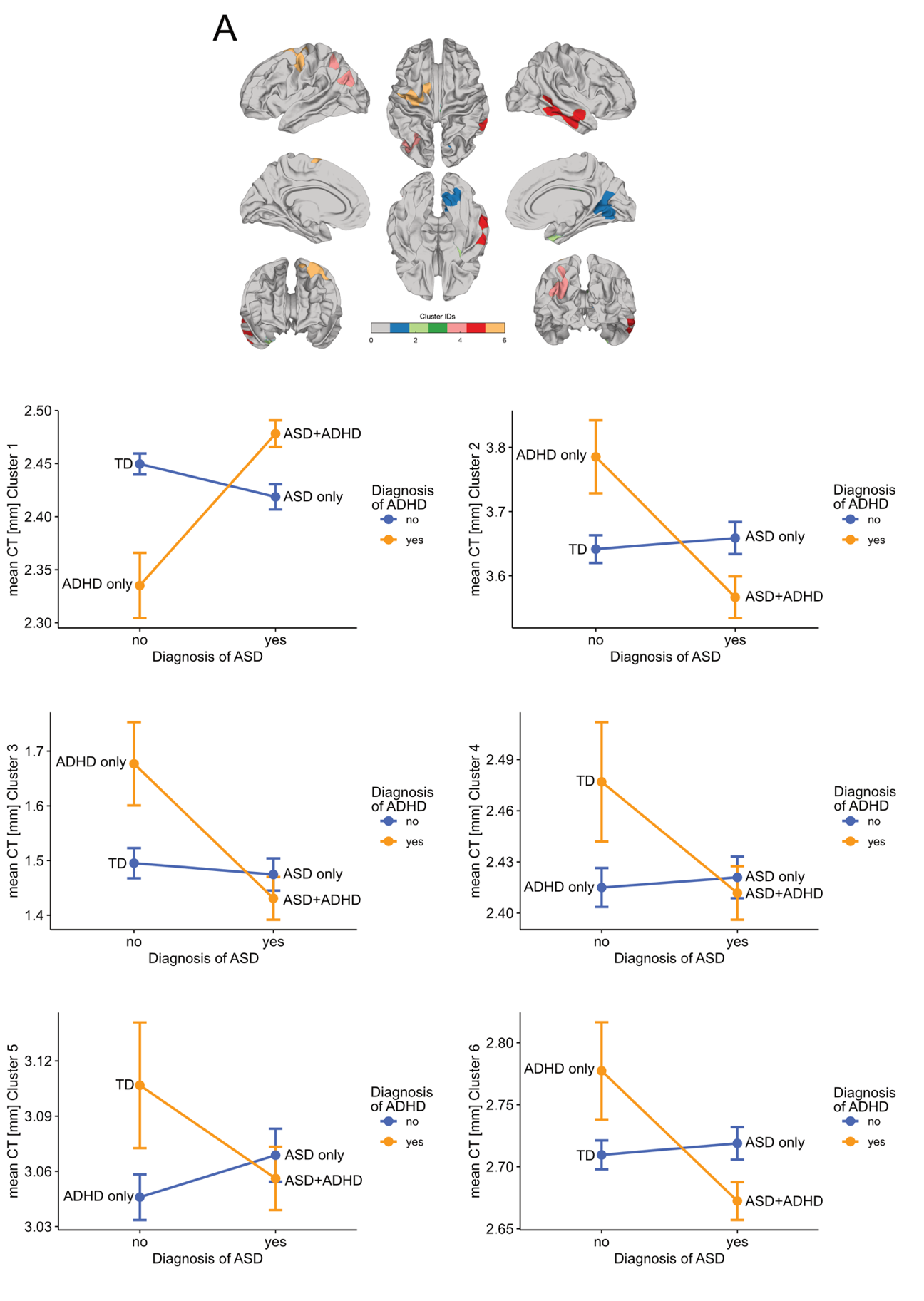


Figure S5A: Interaction plots for all significant clusters for measures of cortical thickness (CT). *Mean CT at significant clusters for each subgroup. Panel A: Number of significant clusters for measures of CT. Note: CT: cortical thickness; ASD: autism spectrum disorder; ADHD: attention-deficit/hyperactivity disorder; TD: typical developing control.*


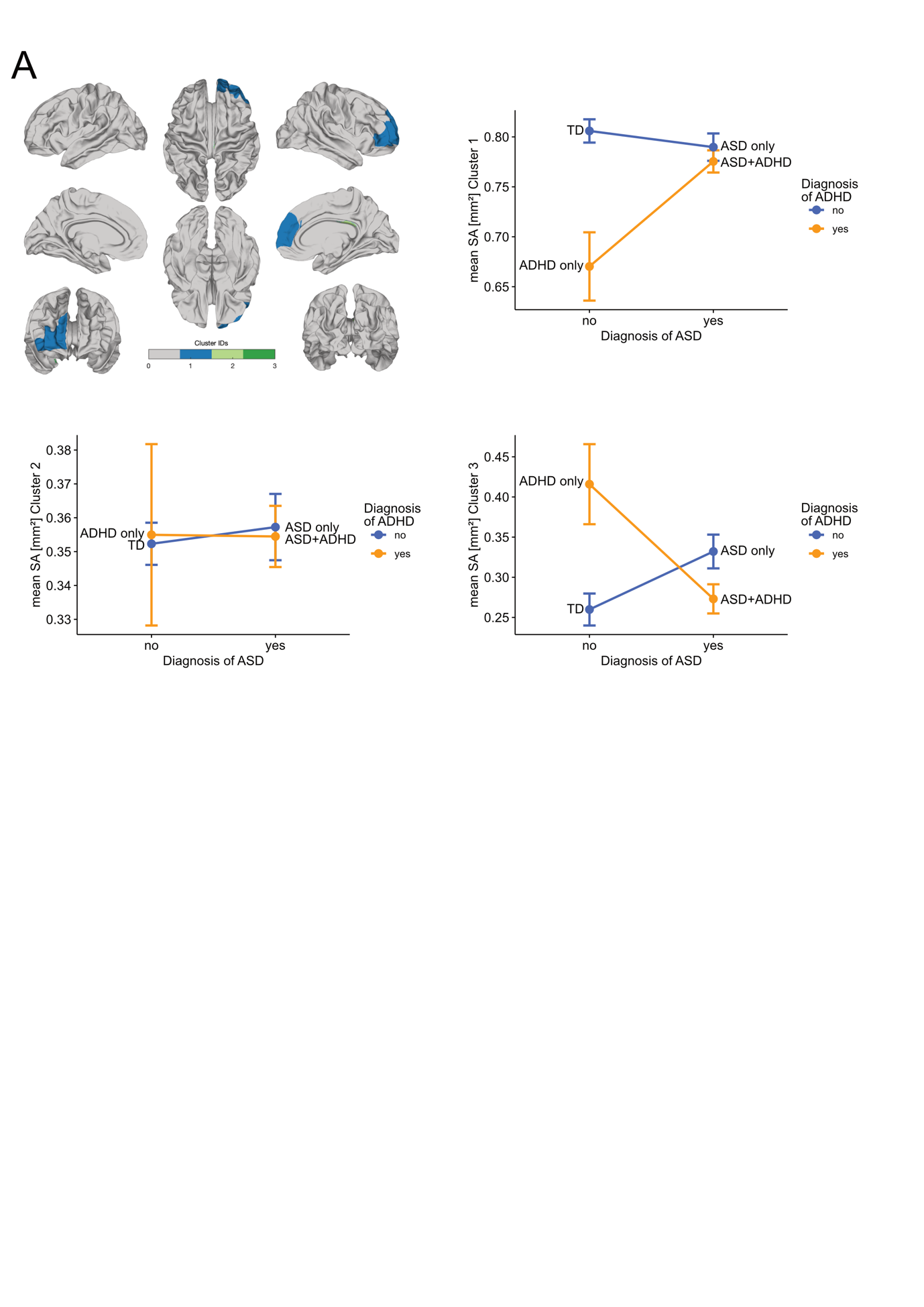


Figure S5B: Interaction plots for all significant clusters for measures of surface area (SA). *Mean SA at significant clusters for each subgroup. Panel A: Number of significant clusters for measures of CT. Note: SA: surface area; ASD: autism spectrum disorder; ADHD: attention-deficit/hyperactivity disorder; TD: typical developing control.*

Figure S6: Gene enrichments as resulting from the GLS-decoding approach. *Panel A and C show the t-maps of the main effect of ASD, the main effect of ADHD and the ASD-by-ADHD interaction term for measures of CT (A) and SA (C). Panel B (CT) and D (SA) show significant odds ratios at a false discovery rate (FDR) corrected p threshold of 0.05 resulting from the gene set enrichment analyses for genes expressed in the different output maps. Gene sets were subdivided into sets with differential gene expression in ASD, sets representing ASD risk genes, and a set representing ADHD risk genes. Gene sets are annotated and labeled based on their original publication. ASD: autism spectrum disorder; ADHD: attention-deficit/hyperactivity-disorder; CTX: cortex; DEG: differentially expressed gene; down: down-regulated expression in ASD; up: upregulated expression in ASD; NGenes: number of genes in each gene set; CT: cortical thickness; SA: surface area; *: p < 0.05 (FDR-corrected), **: p < 0.01 (FDR-corrected); red squares indicate consistently significant enrichments across both approaches (see Figure 4 in main manuscript).*


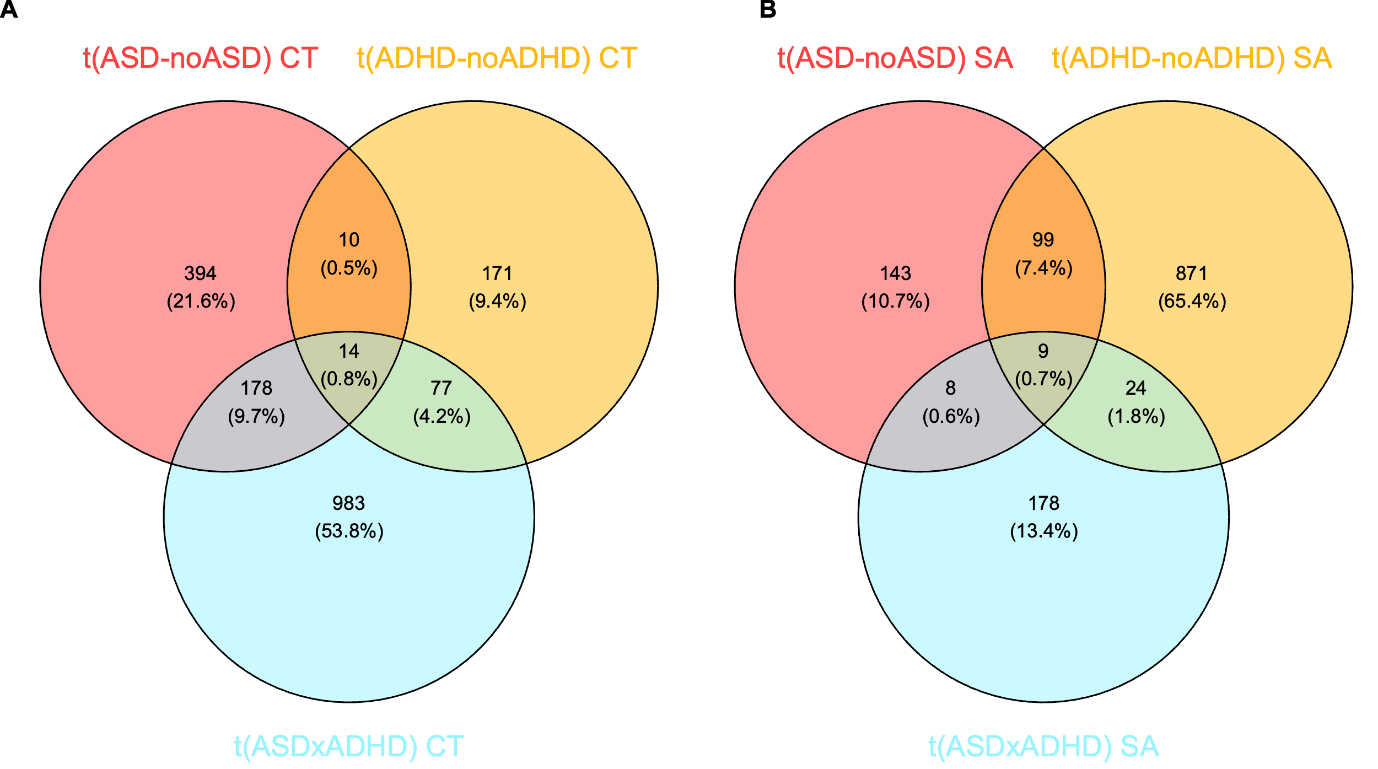


Figure S7: Overlap between significant decoded genes. *Overlap between the genes significantly associated with the main effect of ASD, ADHD, and the interaction term on the transcriptomic level. Panel A shows the overlap between decoded genes for measures of CT. Panel B shows the gene overlap for measures of SA. Note: ASD: autism spectrum disorder; ADHD: attention-deficit/hyperactivity-disorder; CT: cortical thickness; SA: surface area; t: t-statistics.*

# Supplementary Tables

## Table S1:MRI Acquisition Parameters across sites

| **Site** | **Manufacturer** | **Model** | **Software Version** | **Acquisition sequence** | **Coverage** | **Slices** | **Thickness [mm]** | **Resolution [mm^3^]** | **TR [s]** | **TE [ms]** | **FA [°]** | **FOV** |
| --- | --- | --- | --- | --- | --- | --- | --- | --- | --- | --- | --- | --- |
| Cambridge | Siemens | Verio | Syngo MR B17 | Tfl3d1_ns | 256*256 | 176 | 1.2 | 1.1*1.1*1.2 | 2.3 | 2.95 | 9 | 270 |
| London | GS Medical Systems | Discovery mr750 | LX MR DV23.1_V02_1317.c | SAG ADNI GO ACC SPGR | 256*256 | 196 | 1.2 | 1.1*1.1*1.2 | 7.31 | 3.02 | 11 | 270 |
| Mannheim | Siemens | TimTrio | Syngo MR B17 | MPRAGE ADNI | 256*256 | 176 | 1.2 | 1.1*1.1*1.2 | 2.3 | 2.93 | 9 | 270 |
| Nijmegen | Siemens | Skyra | Syngo MRD13 | Tfl3d1_16ns | 256*256 | 176 | 1.2 | 1.1*1.1*1.2 | 2.3 | 2.93 | 9 | 270 |
| Rome | GS Medical Systems | Signa HDxt | 24/LX/MR HD16.0_V02_1131.a | SAG ADNI GO ACC SPGR | 256*256 | 172 | 1.2 | 1.1*1.1*1.2 | 5.96 | 1.76 | 11 | 270 |
| Utrecht | Philips Medical Systems | Achieva/Ingenia CX | 3.2.3,3.2.3.1 | ADNI GO 2 | 256*256 | 170 | 1.2 | 1.1*1.1*1.2 | 6.76 | 3.1 | 9 | 270 |

*Note. TR: repetition time; TE: echo time; FA: flip angle; FOV: flied of view*

## Table S2: Vertex-wise differences in CT and SA for the main effect of ASD

| **Cluster** | | **Regional Labels** | | **Side** | | **BA** | **Vertices** | | **tmax** | **p** | | **Talairach coordinates  x y z** | | | |
| --- | --- | --- | --- | --- | --- | --- | --- | --- | --- | --- | --- | --- | --- | --- | --- |
| **CT** | | | |  | |  |  | |  |  | | |  | | |
| ASD > noASD | | | |  | |  |  | |  |  | | |  | | |
|  | |  | |  | |  |  | |  |  | | |  | | |
| 1 | | rostral anterior cingulate | | L | | 24/33 | 1401 | | 5.27 | 3.43E-06 | | | -4 32 6 | | |
|  | | caudal anterior cingulate cortex | | | |  |  | |  |  | | |  | | |
|  | | superior frontal gyrus | |  | |  |  | |  |  | | |  | | |
| 2 | | superior frontal gyrus | | L | | 21/22 | 6098 | | 3.36 | 4.62E-04 | | | -48 0 -22 | | |
|  | | transverse temporal cortex | |  | |  |  | |  |  | | |  | | |
|  | | banks superior temporal sulcus | |  | |  |  | |  |  | | |  | | |
|  | | middle temporal gyrus | |  | |  |  | |  |  | | |  | | |
| 3 | | superior frontal gyrus | | R | | 4/6/8 | 1850 | | 4.31 | 1.35E-02 | | | 4 32 3 | | |
|  | | caudal anterior cingulate cortex | | | |  |  | |  |  | | |  | | |
| 4 | | precuneus cortex | | R | | 31 | 2610 | | 3.77 | 3.92E-02 | | | 21 -54 19 | | |
|  | | isthmus cingulate cortex | |  | |  |  | |  |  | | |  | | |
|  | | cuneus cortex | |  | |  |  | |  |  | | |  | | |
|  | |  | |  | |  |  | |  |  | | |  | | |
| ASD < noASD | | | |  | |  |  | |  |  | | |  | | |
| 5 | | temporal pole | | R | | 38 | 368 | | -1.65 | 3.94E-06 | | | 32 -1 -22 | | |
| 6 | | superior parietal cortex | | L | | 7 | 7644 | | -1.65 | 5.51E-06 | | | -11-64 51 | | |
|  | | inferior parietal cortex | |  | |  |  | |  |  | | |  | | |
|  | | postcentral gyrus | |  | |  |  | |  |  | | |  | | |
| 7 | | posterior cingulate gyrus | | R | | 23/31 | 44 | | -1.65 | 5.14E-03 | | | 4 -18 27 | | |
| 8 | | caudal middle frontal gyrus | | L | | 6/8/9 | 3337 | | -1.65 | 2.78E-02 | | | -22 12 44 | | |
|  | | rostral middle frontal gyrus | |  | |  |  | |  |  | | |  | | |
|  | | superior frontal gyrus | |  | |  |  | |  |  | | |  | | |
|  | | |  | |  | | |  |  | |  | | |  |  |
| **SA** | | |  | |  | | |  |  | |  | | |  |  |
| ASD < noASD | | |  | |  | | |  |  | |  | | |  |  |
|  |  | |  | |  | | |  |  | |  | | |  |  |
| 1 | anterior cingulate cortex | | | R | 24/33 | | | 1118 | -1.65 | | 1.43E-04 | | | 7 26 22 |  |

*Note. R: right; L: left; BA: approximate Brodmann area(s); Vertices: number of vertices within the cluster; tmax: maximum t-statistic within cluster; p: cluster-corrected p- value.*

## Table S3: Vertex-wise differences in CT and SA for the main effect of ADHD

| **Cluster** | **Regional Labels** | **Side** | | **BA** | | **Vertices** | | **t_max_** | | **p** | | **Talairach coordinates x y z** |
| --- | --- | --- | --- | --- | --- | --- | --- | --- | --- | --- | --- | --- |
| **CT** | |  | |  | |  | |  | |  | |  |
| ADHD < noADHD | |  | |  | |  | |  | |  | |  |
|  |  |  | |  | |  | |  | |  | |  |
| 1 | superior frontal gyrus | R | | 6/8/9 | | 3077 | | -1.65 | | 1.98E-03 | | 6 5 35 |
|  | caudal anterior cingulate gyrus | | |  | |  | |  | |  | |  |
|  | posterior-cingulate cortex |  | |  | |  | |  | |  | |  |
| 2 | lingual gyrus | R | | 31 | | 3489 | | -1.65 | | 4.21E-03 | | 26 -53 6 |
|  | precuneus cortex |  | |  | |  | |  | |  | |  |
|  | cuneus cortex |  | |  | |  | |  | |  | |  |
|  | pericalcarine cortex |  | |  | |  | |  | |  | |  |
| 3 | anterior cingulate cortex | L | | 24/32/33 | | 62 | | -1.66 | | 4.34E-02 | | -5 32 6 |
|  |  |  | |  | |  | |  | |  | |  |
| **SA** |  |  | |  | |  | |  | |  | |  |
| ADHD > noADHD | |  |  | |  | |  | |  | |  | |
|  |  |  |  | |  | |  | |  | |  | |
| 1 | parahippocampal gyrus | L | 27/28 | | 908 | | 3.07 | | 6.22E-03 | | -24 -26 -14 | |

*Note. R: right; L: left; BA: approximate Brodmann area(s); Vertices: number of vertices within the cluster; t_max_: maximum t-statistic within cluster; p: cluster-corrected p- value.*

## Table S4: Vertex-wise differences in CT and SA for the ASD-by-ADHD interaction term

| **Cluster** | **Regional Labels** | **Side** | | **BA** | | | | **Vertices** | **t_max_** | | **p** | **Talairach coordinates x y z** | |
| --- | --- | --- | --- | --- | --- | --- | --- | --- | --- | --- | --- | --- | --- |
| **CT** | |  | | |  | | |  |  | |  |  | |
| Positive interaction* | |  | | |  | | |  |  | |  |  | |
|  |  |  | | |  | | |  |  | |  |  | |
| 1 | lingual gyrus | R | | | 31 | | | 4144 | 5.27 | | 3.45E-04 | 26 -53 6 | |
|  | precuneus cortex |  | | |  | | |  |  | |  |  | |
|  |  |  | | |  | | |  |  | |  |  | |
| Negative interaction* | |  | | |  | | |  |  | |  |  | |
|  |  |  | | |  | | |  |  | |  |  | |
| 2 | entorhinal cortex | R | | | 28/34 | | | 468 | -1.65 | | 3.42E-06 | 27 0 -27 | |
| 3 | posterior cingulate cortex | R | | | 23/24/33 | | | 84 | -1.65 | | 8.99E-06 | 4 -25 26 | |
| 4 | inferior parietal cortex | L | | | 7 | | | 3893 | -1.65 | | 5.32E-03 | -26 -51 48 | |
|  | superior parietal cortex |  | | |  | | |  |  | |  |  | |
| 5 | middle temporal gyrus | R | | | 20/21/22 | | | 3479 | -1.65 | | 2.69E-02 | 59 -39 -10 | |
|  | inferior temporal gyrus |  | | |  | | |  |  | |  |  | |
|  | banks superior temporal sulcus |  | | |  | | |  |  | |  |  | |
|  | superior temporal gyrus |  | | |  | | |  |  | |  |  | |
| 6 | precentral gyrus | L | | | 6/8 | | | 3614 | -1.65 | | 3.16E-02 | -45 -10 41 | |
|  | superior frontal gyrus |  | | |  | | |  |  | |  |  | |
|  |  |  | | |  | | |  |  | |  |  | |
| **SA** |  |  | | |  | | |  |  | |  |  | |
| Positive interaction* | | |  | | |  |  | | |  |  | |  |
|  |  | |  | | |  |  | | |  |  | |  |
| 1 | superior frontal gyrus | | R | | | 46 | 7974 | | | 4.25 | 2.22E-02 | | 10 59 6 |
|  | rostral middle frontal gyrus | |  | | |  |  | | |  |  | |  |
|  | pars triangularis | |  | | |  |  | | |  |  | |  |
|  | pars orbitalis | |  | | |  |  | | |  |  | |  |
|  |  | |  | | |  |  | | |  |  | |  |
| Negative interaction* | | |  | | |  |  | | |  |  | |  |
|  |  | |  | | |  |  | | |  |  | |  |
| 2 | posterior cingulate gyrus | | R | | | 23/31 | 322 | | | -1.65 | 5.15E-06 | | 4 -30 25 |
| 3 | lingual gyrus | | R | | | 19/30 | 29 | | | -1.67 | 1.08E-02 | | 24 -19 -19 |

*Note. R: right; L: left; BA: approximate Brodmann area(s); Vertices: number of vertices within the cluster; t_max_: maximum t-statistic within cluster; p: cluster-corrected p- value. * For interaction plots of each cluster see Figure S5A (CT) and S5B (SA).*

## Table S5: Vertex-wise differences in CT for the main effect of ASD+ADHD

| **Cluster** | **Regional Labels** | **Side** | **BA** | **Vertices** | **t_max_** | **p** | **Talairach coordinates x y z** |
| --- | --- | --- | --- | --- | --- | --- | --- |
| ASD+ADHD < ASD only | |  |  |  |  |  |  |
|  |  |  |  |  |  |  |  |
| 1 | precentral gyrus | L | 6/8/9 | 3427 | -1.65 | 4.66E-02 | -50 -6 42 |
|  | caudal middle frontal gyrus |  |  |  |  |  |  |
|  | postcentral gyrus |  |  |  |  |  |  |

*Note. R: right; L: left; BA: approximate Brodmann area(s); Vertices: number of vertices within the cluster; t_max_: maximum t-statistic within cluster; p: cluster-corrected p- value.*

# References

1. Lord C, Risi S, Lambrecht L, Cook EH, Leventhal BL, DiLavore PC, et al. The autism diagnostic observation schedule-generic: a standard measure of social and communication deficits associated with the spectrum of autism. J Autism Dev Disord [Internet]. 2000 Jun;30(3):205–23. Available from: http://www.ncbi.nlm.nih.gov/pubmed/11055457

2. Rutter M, Le Couteur A, Lord C. Autism diagnostic interview-revised. Los Angeles, CA: Western Psychological Services. 2003;29(2003):30.

3. American Psychiatric Association (APA). Diagnostic and Statistical Manual of Mental Disorders (DSM-5®). 2013;

4. Radua J, Vieta E, Shinohara R, Kochunov P, Quidé Y, Green MJ, et al. Increased power by harmonizing structural MRI site differences with the ComBat batch adjustment method in ENIGMA. Neuroimage [Internet]. 2020 Sep;218:116956. Available from: https://linkinghub.elsevier.com/retrieve/pii/S1053811920304420

5. Ecker C, Ronan L, Feng Y, Daly E, Murphy C, Ginestet CE, et al. Intrinsic gray-matter connectivity of the brain in adults with autism spectrum disorder. Proc Natl Acad Sci U S A. 2013;110(32):13222–7.

6. Fulcher BD, Arnatkeviciute A, Fornito A. Overcoming false-positive gene-category enrichment in the analysis of spatially resolved transcriptomic brain atlas data. Nat Commun [Internet]. 2021;12(1):1–13. Available from: http://dx.doi.org/10.1038/s41467-021-22862-1

7. Markello RD, Arnatkeviciute A, Poline JB, Fulcher BD, Fornito A, Misic B. Standardizing workflows in imaging transcriptomics with the abagen toolbox. Elife [Internet]. 2021 Nov 16;10. Available from: https://elifesciences.org/articles/72129

8. Postelnicu G, Zollei L, Fischl B. Combined volumetric and surface registration. IEEE Trans Med Imaging [Internet]. 2009 Apr;28(4):508–22. Available from: http://www.ncbi.nlm.nih.gov/pubmed/19273000

# The EU-AIMS LEAP consortium:

Jumana Ahmad^5,13^, Sara Ambrosino^14^, Bonnie Auyeung^15,16^, Tobias Banaschewski^8^, Simon Baron-Cohen^16^, Sarah Baumeister^8^, Christian F. Beckmann^6^, Sven Bölte^17,18,19^, Thomas Bourgeron^12^, Carsten Bours^6^, Michael Brammer^5^, Daniel Brandeis^8,20,21^, Claudia Brogna^22^, Yvette de Bruijn^6^, Jan K. Buitelaar^6^, Bhismadev Chakrabarti^16^, Tony Charman^9^, Ineke Cornelissen^6^, Daisy Crawley^5^, Flavio Dell’Acqua^5^, Guillaume Dumas^12^, Sarah Durston^10^, Christine Ecker^1,2,5^, Jessica Faulkner^5^, Vincent Frouin^23^, Pilar Garcés^24^, David Goyard^23^, Lindsay Ham^25^, Hannah Hayward^5^, Joerg Hipp^24^, Rosemary Holt^16^, Mark H. Johnson^10^, Emily J.H. Jones^10^, Prantik Kundu^26^, Meng-Chuan Lai^27^, Xavier Liogier D’Ardhuy^24^, Michael V. Lombardo^28^, Eva Loth^5^, David J. Lythgoe^29^, René Mandl^30^, Andre Marquand^6^, Luke Mason^10^, Maarten Mennes^6^, Andreas Meyer-Lindenberg^8^, Carolin Moessnang^8^, Nico Bast^1^, Declan G.M. Murphy^5^, Bethany Oakley^5^, Laurence O’Dwyer^6^, Marianne Oldehinkel^6^, Bob Oranje^10^, Gahan Pandina^31^, Antonio M. Persico^32^, Barbara Ruggeri^33^, Amber Ruigrok^16^, Jessica Sabet^5^, Roberto Sacco^22^, Antonia San José Cáceres^5^, Emily Simonoff^34^, Will Spooren^24^, Julian Tillmann^11^, Roberto Toro^12^, Heike Tost^8^, Jack Waldman^16^, Steve C.R. Williams^29^, Caroline Wooldridge^29^, and Marcel P. Zwiers^6^.

^1^ Department of Child and Adolescent Psychiatry, University Hospital, Goethe University, Deutschordenstrasse 50, 60528 Frankfurt am Main, Germany.

^2^ Brain Imaging Center, Goethe University, 60528 Frankfurt am Main, Germany.

^5^ Department of Forensic and Neurodevelopmental Sciences, Institute of Psychiatry, Psychology and Neuroscience, King’s College, London SE5 8AF, UK.

^6^ Department of Cognitive Neuroscience, Donders Institute for Brain, Cognition and Behaviour, Radboud University Nijmegen Medical Center, Nijmegen, The Netherlands.

^8^ Child and Adolescent Psychiatry, Central Institute of Mental Health, University of Heidelberg, Medical Faculty Mannheim, Mannheim, Germany.

^9^ Department of Psychology, Institute of Psychiatry, Psychology and Neuroscience, King’s College London, London SE5 8AF, UK.

^10^ Centre for Brain and Cognitive Development, Birkbeck, University of London, Malet Street, London WC1E 7JL, UK.

^11^ F. Hoffmann–La Roche, Innovation Center Basel, Basel, Switzerland.

^12^ Institut Pasteur, Human Genetics and Cognitive Functions Unit, Paris, France.

^13^ Department of Psychology, Social Work and Counselling, Faculty of Education and Health, Greenwich University, London, UK.

^14^ University Medical Center Utrecht, Utrecht University, Utrecht, Netherlands.

^15^ School of Philosophy, Psychology and Language Sciences, University of Edinburgh, 7 George Square, Edinburgh EH8 9JZ, UK

^16^ Autism Research Centre, Department of Psychiatry, University of Cambridge, Cambridge, UK

^17^ Center of Neurodevelopmental Disorders (KIND), Centre for Psychiatry Research, Department of Women’s and Children’s Health, Karolinska Institutet & Stockholm Health Care Services, Region Stockholm, Stockholm, Sweden

^18^ Child and Adolescent Psychiatry, Stockholm Health Care Services, Region Stockholm, Stockholm, Sweden

^19^ Curtin Autism Research Group, Curtin School of Allied Health, Curtin University, Perth, Australia

^20^ Department of Child and Adolescent Psychiatry and Psychotherapy, Psychiatric Hospital, University of Zurich, Zurich, Switzerland.

^21^ Neuroscience Center Zurich, University and ETH Zurich, Zurich, Switzerland.

^22^ Pediatric Neurology Unit, Università Cattolica del Sacro Cuore, 00168 Rome, Italy.

^23^ Neurospin Centre CEA; Gif sur Yvette, France.

^24^ Roche Pharma Research and Early Development, Neuroscience, Ophthalmology and Rare Diseases, Roche Innovation Center Basel; Basel, Switzerland.

^25^ Regulatory Affairs, Pharmaceutical Development, F. Hoffmann-La Roche Pharmaceuticals; Basel, Switzerland.

^26^ Department of Radiology, Icahn School of Medicine at Mount Sinai; New York, New York, USA

^27^ Child and Youth Mental Health Collaborative, Centre for Addiction and Mental Health and The Hospital for Sick Children, Department of Psychiatry, University of Toronto; Toronto, Canada.

^28^ Laboratory for Autism and Neurodevelopmental Disorders, Center for Neuroscience and Cognitive Systems @UniTn, Istituto Italiano di Tecnologia, Rovereto, Italy.

^29^ Department of Neuroimaging, Institute of Psychiatry, Psychology and Neuroscience, King's College London; London, United Kingdom.

^30^University Medical Center Utrecht, Utrecht University, Utrecht, Netherlands.

^31^ Janssen Research & Development; Titusville, New Jersey, USA.

^32^ Child and Adolescent Neuropsychiatry, Department of Biomedical, Metabolic and Neural Sciences, University of Modena and Reggio Emilia, Modena, Italy.

^33^ Social, Genetic and Developmental Psychiatry Centre, Institute of Psychiatry, Psychology and Neuroscience, King's College London; London, United Kingdom

^34^ Department of Child and Adolescent Psychiatry, Institute of Psychology, Psychiatry and Neuroscience, King's College London; London, United Kingdom.
